# Physiology of Highly Radioresistant *Escherichia coli* After Experimental Evolution for 100 Cycles of Selection

**DOI:** 10.3389/fmicb.2020.582590

**Published:** 2020-09-22

**Authors:** Steven T. Bruckbauer, Joel Martin, Benjamin B. Minkoff, Mike T. Veling, Illissa Lancaster, Jessica Liu, Joseph D. Trimarco, Brian Bushnell, Anna Lipzen, Elizabeth A. Wood, Michael R. Sussman, Christa Pennacchio, Michael M. Cox

**Affiliations:** ^1^Department of Biochemistry, University of Wisconsin-Madison, Madison, WI, United States; ^2^DOE Joint Genome Institute, Berkeley, CA, United States; ^3^Center for Genomic Science Innovation, University of Wisconsin School of Medicine and Public Health, Madison, WI, United States; ^4^Department of Systems Biology, Harvard Medical School, Boston, MA, United States

**Keywords:** ionizing radiation, experimental evolution, reactive oxygen species, double-strand breaks, DNA repair, *Escherichia coli*, *Deinococcus radiodurans*

## Abstract

Ionizing radiation (IR) is lethal to most organisms at high doses, damaging every cellular macromolecule via induction of reactive oxygen species (ROS). Utilizing experimental evolution and continuing previous work, we have generated the most IR-resistant *Escherichia coli* populations developed to date. After 100 cycles of selection, the dose required to kill 99% the four replicate populations (IR9-100, IR10-100, IR11-100, and IR12-100) has increased from 750 Gy to approximately 3,000 Gy. Fitness trade-offs, specialization, and clonal interference are evident. Long-lived competing sub-populations are present in three of the four lineages. In IR9, one lineage accumulates the heme precursor, porphyrin, leading to generation of yellow-brown colonies. Major genomic alterations are present. IR9 and IR10 exhibit major deletions and/or duplications proximal to the chromosome replication terminus. Contributions to IR resistance have expanded beyond the alterations in DNA repair systems documented previously. Variants of proteins involved in ATP synthesis (AtpA), iron-sulfur cluster biogenesis (SufD) and cadaverine synthesis (CadA) each contribute to IR resistance in IR9-100. Major genomic and physiological changes are emerging. An isolate from IR10 exhibits protein protection from ROS similar to the extremely radiation resistant bacterium *Deinococcus radiodurans*, without evident changes in cellular metal homeostasis. Selection is continuing with no limit to IR resistance in evidence as our *E. coli* populations approach levels of IR resistance typical of *D. radiodurans*.

## Introduction

Ionizing radiation (IR) is a source of severe oxidative stress to organisms. IR-generated reactive oxygen species (ROS), particularly hydroxyl radicals, will cause oxidative damage to the cell membrane, proteome, and genome (Ruiz de Almodovar et al., 1994; Kempner, 2001; Harris et al., 2009; Daly, 2012; Reisz et al., 2014; Cao et al., 2015; Bruckbauer et al., 2020). DNA double-strand breaks (DSB) are a potent form of damage caused by IR, as a single unrepaired DSB is lethal to a cell. With increasing doses of IR, cells must contend with ever-worsening ROS stress and DNA damage.

There are no natural environments that feature high levels of ionizing radiation, yet radioresistant organisms are present in all kingdoms of life. Perhaps the most well-studied of these extremophiles is the bacterium *Deinococcus radiodurans* (Cox and Battista, 2005; Daly, 2009; Slade and Radman, 2011). Accumulation of cytosolic Mn^2+^ affords *D. radiodurans* enhanced amelioration of ROS generated by IR (Daly et al., 2004, 2010; Sharma et al., 2017). Furthermore, this bacterium encodes an array of unique DNA repair enzymes that work in concert with standard DNA repair mechanisms which allow for efficient repair of hundreds of IR-induced DNA DSBs (Earl et al., 2002; Harris et al., 2004; Tanaka et al., 2004; Cox and Battista, 2005; Selvam et al., 2013). With these mechanisms, *D. radiodurans* can survive doses of IR well over 10 kGy. In at least some cases, extreme IR resistance is associated with adaptation for extreme desiccation resistance (Tanaka et al., 2004; Cox and Battista, 2005). Upon rehydration after extended desiccation, adapted bacteria can rapidly repair damaged DNA, replenish the proteome, and return to growth.

Experimental evolution in the laboratory has been utilized in many previous efforts to generate IR resistance in bacteria. Early work on IR resistance was conducted prior to DNA sequencing technologies which allow for rapid genotyping of mutants and were thus unable to directly link resistance with causative mutations (Witkin, 1946; Erdman et al., 1961; Davies and Sinskey, 1973; Parisi and Antoine, 1974). We have embarked on a long-term effort to evolve and characterize laboratory-generated IR-resistance in the model bacterium *Escherichia coli*.

Our first effort focused on four replicate populations of *E. coli* which were selected for radioresistance over 20 iterative cycles of exposure to gamma ray IR emitted by 60Co (Harris et al., 2009; Byrne et al., 2014). This work succeeded in generating substantial increases in radiation resistance. The IR resistance phenotype was attributed largely to alterations in DNA repair pathways, although a few other contributions were apparent (Harris et al., 2009; Byrne et al., 2014; Bruckbauer et al., 2019a,b). A continuation of that effort proved impossible due to ongoing 60Co source decay and required irradiator de-commissioning. To carry out a long-term evolution trial with a new radiation source, we initiated a second trial. The IR source of choice was a Varian 21EX clinical linear accelerator (Linac), generating high energy electron beam IR at a dose rate nearly four times higher than that of the previous study (Bruckbauer et al., 2019b). Source decay was no longer a problem.

The second effort again involved four replicate populations of *E*. *coli*, this time challenged with Linac irradiation at 72 Gy/min. This ongoing evolution trial has already generated far more resistant *E. coli* populations than those produced previously. Furthermore, recent advances in sequencing technologies have allowed for whole-genome sequencing of entire populations throughout the cycles of selection, illuminating the underlying genetic complexities of experimental evolution. Both in the original evolution trial and in the first 50 selection cycles of the new one, the first prominent genetic alterations conferring IR resistance centered on mutations modifying or eliminating the function of particular DNA repair enzymes (Harris et al., 2009; Byrne et al., 2014; Bruckbauer et al., 2019b).

We now present the next step in the long-term evolution of IR resistance experiment. We have continued evolution of the four replicate lineages of IR-resistant *E. coli* for 50 further cycles of selection. At round 100 of selection, a dose of 3,000 Gy is required to kill 99% of cells in these populations. At a dose of 3,000 Gy, survival is increased approximately 100-fold relative to the same lineages after 50 cycles of selection. Isolates from these evolved populations exhibit a wide array of genetic and phenotypic differences, as these evolved *E. coli* have begun to diverge significantly both between and within populations. In contrast to each of the previous evolution trials, mechanisms of IR resistance have expanded well beyond modifications to DNA repair mechanisms.

## Materials and Methods

### Data Availability

All mutations called (in reference to the MG1655 U00096.3 genome) in the population and isolate sequencing data are readily accessible in Supplementary Dataset 1 and Supplementary Dataset 2. All raw mass spectrometry data is publicly available on the Chorus project under Project #1688.^1^ Processed mass spectrometry data is accessible in Supplementary Dataset 3. All population and isolate sequencing data generated by the Joint Genome Institute is available through the National Center for Biotechnology Information (NCBI) Sequencing Read Archive (SRA).^2^ All SRA accession numbers are listed in Supplementary Dataset 4.

### Growth Conditions and Bacterial Strains Used in This Study

Unless otherwise stated, *E. coli* cultures were grown in Luria-Bertani (LB) broth (Miller, 1992) at 37°C with aeration. *E. coli* were plated on 1.5% LB agar medium (Miller, 1992) and incubated at 37°C. Overnight cultures were grown in a volume of 3 mL for 16 to 18 h. Exponential phase cultures were routinely prepared by diluting overnight cultures 1:100 in 10 mL of LB medium in a 50 mL Erlenmeyer flask and were grown at 37°C with shaking at 200 rpm and were harvested at an OD_600_ of 0.2 (early exponential phase), unless otherwise noted. After growth to an OD_600_ of 0.2, cultures were placed on ice for 10 min to stop growth before being used for assays.

All strains used for *in vivo* assays in this study are mutants of *E. coli* K-12 derivative MG1655. Genetic manipulations to transfer mutations or delete genes were performed as previously described (Datsenko and Wanner, 2000; Warming et al., 2005). Strains used in this study are listed in Supplementary Table S1.

### Serial Dilutions and CFU/mL Determination

All serial dilutions were performed in 1X phosphate-buffered saline (PBS) (for 1 L: 8 g NaCl, 0.2 g KCl, 1.44 g Na_2_HPO_4_, KH_2_PO_4_ 0.24 g with 800 mL dH_2_O, adjust pH with HCl to 7.4, then add remaining 200 mL dH_2_O**).** Unless otherwise stated, serial dilutions were performed with serial 1:10 dilutions of 100 μL of culture or previous dilution into 900 μL 1X PBS. Before transfer to the next dilution tube, samples were vortexed for 2 seconds and mixed by pipetting to ensure mixing. One-hundred μL of appropriate dilutions were aliquoted onto agar plates of the appropriate medium and were spread-plated utilizing an ethanol-sterilized, bent glass rod. For spot plating, 10 μL of each dilution was aliquoted onto agar plates of the appropriate medium and spots were allowed to dry before plates were incubated as in *Growth conditions*.

CFU/mL was calculated using the highest CFU count for each strain assayed that remained between 30 and 300 CFU (ex: 250 CFU on a 10^–4^ dilution plate would be used for calculation over 40 CFU on a 10^–5^ dilution plate).

### Generalized Linac Irradiation Protocol

Samples were maintained at 4°C using a cold block (Corning Inc.; Corning, NY, United States; Cat #: 432041) and transported to the University of Wisconsin Medical Radiation Research Center (UWMRRC) Varian 21EX clinical linear accelerator (Linac) facility for irradiation. The total transport time was approximately 15 min to and from the Linac facility. For each irradiation, the Linac was set to deliver a beam of electrons with 6 MeV of energy to uniformly irradiate all samples (a total of 14) at once. To accomplish this, a special high-dose mode called HDTSe^–^ was utilized, which resulted in a dose rate to the samples of approximately 72 Gy/min. The sample tubes were placed horizontally and submerged at a depth of 1.3 cm (measured to the center of the tube’s volume) in an ice-water filled plastic tank, where water was replaced every 2,000 Gy (as necessary) to maintain a temperature of 4 – 10°C. Samples were set to a source-to-surface distance (SSD) of 61.7 cm. A 30 × 30 cm^2^ square field size was set at the Linac console, which gave an effective field size at this SSD of 18.5 × 18.5 cm^2^. This is ample coverage to provide a uniform dose to all of the sample vials. The monitor unit calculations (determination of the amount of time to leave the Linac on) were based on the American Association of Physicists in Medicine (AAPM) Task Group 51 protocol for reference dosimetry (Almond et al., 1999). This is the standard method for determining dose per monitor unit in water for radiation therapy calculations. Once the dose was determined in the AAPM Task Group 51 reference protocol conditions (SSD = 100 cm and depth = 10 cm), an ion chamber and water-equivalent plastic slabs were used to translate this dose to the specific conditions used in this project. An independent dose verification was performed with thermoluminescent dosimeters (TLDs) (Bruckbauer et al., 2019b). TLDs are passive dosimeters that are small, accurate and well-suited for dose verification in the routinely used 1.5 mL sample vials.

### Ionizing Radiation Resistance Assay Using the Linac

Strains were grown in biological triplicate overnight and to an OD_600_ of approximately 0.2 in LB as routinely performed. A 1 mL sample for each dose tested (including 0 Gy) was removed and aliquoted into a sterile 1.5 mL microfuge tube. Samples were pelleted by centrifugation at 13,000 x*g* for 1 min, and the supernatant was poured off. Samples were resuspended in 1 mL ice-cold 1X phosphate-buffered saline (PBS), and pelleting was repeated. This process was repeated three more times to wash cells. A 100 μL aliquot of each culture was removed, serial diluted 1:10 in 900 μL of PBS to a final 10,000-fold dilution and 100 μL was plated on LB agar to determine the colony forming units (CFU)/mL before irradiation. Samples were maintained at 4°C and irradiated with the appropriate doses as described. A 100 μL aliquot of each culture was removed and plated to determine CFU/mL and percent survival as described.

### Directed Evolution Protocol Using Linac

For each round of directed evolution, separate aliquots of 2 mL of LB medium was inoculated with frozen stock of each population from the previous round of selection. These were incubated overnight with aeration at 37°C and were grown with usual practices in LB medium to an OD_600_ of 0.2 the next day. Each culture was incubated on ice for 10 min to stop growth. Three 1 mL samples were removed and aliquoted into sterile 1.5 mL microfuge tubes. Samples were washed three times with 1 mL ice-cold 1X phosphate-buffered saline (PBS) and resuspended in a final volume of 1 mL of 1X PBS. A 100 μL aliquot of each culture was removed, serial diluted 1:10 in 900 μL of PBS to a final 10,000-fold dilution and 100 μL was plated on LB agar to determine the colony forming units (CFU)/mL before irradiation. Samples were maintained at 4°C and taken to a Varian 21EX clinical linear accelerator (Linac) for irradiation.

After irradiation, an aliquot of each culture was removed, serial diluted 1:10 in 900 μL of PBS to a final 1,000-fold dilution, and 100 μL of each serial dilution was plated on LB agar for each dose to determine the CFU/mL after irradiation. LB agar plates were incubated overnight at 37°C. Remaining irradiated cultures were pelleted by centrifugation at 13,000 x*g* and supernatant was discarded. These pellets were resuspended in 1 mL of fresh LB medium, and this was added to 1 mL LB medium in a 5 mL glass culture tube. These resuspensions were incubated overnight with aeration at 37°C. The following day, the percent survival for each dose was calculated using CFU/mL calculations before and after irradiation at each dose. The overnight culture of each population replicate showing closest to 1% survival was stored at -80°C and used for the next cycle of selection. One cycle of selection was performed weekly due to limited access to the Linac.

The initiating round of selection was done as described above, except the original culture used was an overnight culture of MG1655 prepared from an isolated colony. This protocol was adapted from a previously used protocol (Harris et al., 2009).

### Growth Curves

Strains were cultured described as in *Growth conditions* overnight and to an OD_600_ of 0.2 in LB medium, EZ medium supplemented with 0.2% glucose (Teknova; Hollister, CA, United States) and M9 minimal medium (Miller, 1992) supplemented with 0.2% glucose. Cultures were then diluted 1:100 in the appropriate medium in a clear, flat bottom 96-well plate (Fisher product #: 07-200-656) and incubated overnight in a Biotek Synergy 2 plate reader (Biotek; Winooski, VT, United States) at 37°C with shaking, with OD_600_ readings taken by the plate reader every 10 min.

### Resistance to DNA-Damaging Agents

Cells were grown overnight and to an OD_600_ of approximately 0.2 in LB as routinely performed. Samples were mixed by vortexing for 5 s and were serial diluted 1:10 in 900 μl phosphate-buffered saline (PBS) to a final 100,000-fold dilution. Ten μl was removed from each dilution and spotted onto 30 mL 1.5% LB agar medium supplemented with 10 or 7.5 ng/ml ciprofloxacin hydrochloride as specified, 4 μg/mL mitomycin C, or 5 mM hydroxyurea. Spots were dried before being incubated overnight at 37°C. Plates were imaged after 48 h for all plates unless stated otherwise.

### UV Resistance Assay

Cells from a single colony of each strain were cultured overnight and then grown to an OD_600_ of ∼ 0.2 as in *Growth conditions.* Samples were diluted and spotted onto 25 mL 1.5% LB agar described in the *Serial dilutions.* Spots were dried before the plate lid was removed and spots were exposed to the appropriate dose of UV irradiation using a Spectrolinker XL-1000 UV Crosslinker (Spectronics Corporation, Westbury, NY, United States). Plates were imaged after incubation for 24 h.

### Desiccation Tolerance Assay

The bacterial desiccation tolerance assay was performed similarly to a previously reported protocol (Boothby et al., 2017). *E. coli* strains were struck out on LB agar medium with no antibiotic (BD Biosciences, San Jose, CA, United States; Cat #: 240110) and allowed to grow at 37°C overnight. Nine single colonies were picked and inoculated into 3 mL of liquid LB medium with no antibiotics (BD Biosciences, San Jose, CA, United States; Cat #: 240230). These were grown for 16 h with at 37°C with 220 RPM circular agitation in a Multitron Infors HT shaker incubator. The OD_600_ of the sample was measured using a WPA Biowave Cell Density Meter CO8000. The approximate number of cells per mL was calculated using the conversion factor 7.00 × 10^8^ (cells/mL)/(OD_600_) (Sezonov et al., 2007). Based on this calculation, 1.00 × 10^8^ cells were transferred into a 1.7 mL microcentrifuge tube. This tube was spun at 10,000 Xg for 5 min at room temperature. Medium was carefully removed as not to disturb the bacterial pellet. The pellet was resuspended in 1 mL of PBS. 1 μL of this was used to prepare a 1:1000 dilution in PBS (theoretical 1.00 × 10^5^ cells/mL). This dilution was plated with an Eddy Jet 2W - Spiral Plater to calculate CFU/mL of the starting material.

The remaining sample was spun at 10,000 X g for 5 min in at room temperature to re-pellet the sample. The PBS was carefully removed with a pipette. The pellets were then placed in a Thermo Fisher speedvac concentrator (Thermo-Fisher, Waltham, MA, United States; Cat#: SPD111V-115) connected to an HFS vp2200 vacuum pump. Samples were dried in this speedvac for 24 h. The next day, samples were either directly resuspended in 1 mL of SOC (overnight drying) or transferred to a vacuum desiccator for a week (1 week drying) before being resuspended in 1 mL of SOC. The CFUs/mL were then calculated using the Eddy Jet 2W – Spiral Plater system. To calculate survival rates, the CFU/mL of the cells post drying were normalized to matched input CFU/mL. Survival rates were calculated for a total of nine biological replicates for each of the reported strains.

### Metals Analysis

Cultured appropriate strains in biological triplicates as described in ‘*Growth conditions*’ to an OD_600_ of 0.2. One mL of each was washed 3 times in 1X Dulbecco’s PBS (Sigma-Aldrich, St. Louis, MO, United States; Cat #: D1283) as described in ‘*Ionizing radiation resistance assay.*’ Store-bought PBS was used to avoid the possibility of metals contamination from lab-specific dH_2_O. One-hundred μL of each sample was diluted and plated as in ‘*Serial dilutions*’ in order to determine the CFU/mL of each sample to normalize relative amounts of quantified elements to cell concentration. The remaining washed samples were transported at 4°C to the University of Wisconsin State Laboratory of Hygiene Trace Element Research Laboratory for Magnetic Sector inductively coupled plasma mass spectrometer (ICPMS) analysis.

### β-Galactosidase Assay

The β-galactosidase assay was adapted from a previously described protocol (Bruckbauer et al., 2019a). Cultures to be assayed were prepared by incubating cells from a single colony of each strain overnight at 37°C with aeration. This resulting overnight culture (grown 15-18 h) was treated as the stationary phase culture to be assayed. Exponential phase cultures were grown by inoculating 10 mL of LB broth in a 50 mL Erlenmeyer flask with 70 μL of overnight culture and grown at 37°C with shaking to an OD_600_ 0.2. These cultures were placed on ice for at least 5 min to stop growth before use. To perform a mock irradiation, two separate aliquots of 900 μL of exponential phase cultures in 1.5 mL Eppendorf tubes were inverted and incubated in the dark at room temperature (∼24°C) for 8 h. Two aliquots for each replicate ensures that there is sufficient volume for 1 mL of culture to determine β-galactosidase activity and 100 μL to determine OD_600_.

The β-galactosidase assay was carried out as follows. An OD_600_ reading was taken of each culture, and an appropriate amount (1 mL for exponential phase and mock irradiation cultures, and 50 μL for stationary phase) was aliquoted into 2 mL microcentrifuge tubes. Cells were pelleted via centrifugation at 6900 x*g* for 3 min, and supernatant was removed. Cells were resuspended in 1 mL Z buffer (0.06 M Na_2_HPO_4_, 0.04 NaH_2_PO4, 0.01 KCl, 0.001M MgSO_4_, to volume with purified dH_2_O), and 1 mL Z buffer was aliquoted in a 2 mL microcentrifuge tube for a blank sample. One-hundred μL chloroform and 50 μL 0.1% SDS were added to each tube. Each sample was then vortexed for 10 s and incubated at 4°C for at least 10 min. Three samples at a time were removed from 4°C and placed in a 28°C water bath for 5 min. Two-hundred μL 4 mg/mL O-Nitrophenyl β-D-Galactopyranoside (ONPG) (Sigma-Aldrich, St. Louis, MO, United States Cat#: N1127) dissolved in Z buffer was added to each sample. The development of yellow coloration for each sample was timed. Once the sample had become yellow (or after 30 min) the reaction was stopped by adding 500 μL 1M Na_2_CO_3_ and samples were placed on ice. All samples were spun for 10 min at 17000 xg in a microcentrifuge at 4°C. One mL was removed from each sample, and the OD_420_ and OD_550_ was read. To determine β-galactosidase activity, the following equation was used where *t* is time of the reaction and *v* is the volume of culture used:

$$A\,c\,t\,i\,v\,i\,t\,y=\frac{1000*\left(\left(O\,D_{420}-\left(1.75*O\,D_{550}\right)\right)\right)}{t*v*O\,D_{600}}$$

All β-galactosidase assays were carried out using biological triplicate. To determine relative β-galactosidase activity compared to the parent strain, the activity of each mutant strain replicate was divided by the average activity of the wild-type triplicate in the given experiment.

### SOS Response Assay

After irradiation (see *Ionizing radiation resistance assay using the Linac*), mock-treated and irradiated cultures were diluted 1:100 in the appropriate medium, and then aliquoted into a black-walled, flat bottom 96-well plate (Corning, Corning, NY, United States; Cat #: 3916) and incubated overnight in a Biotek Synergy 2 plate reader, with OD_600_ measurements and Ex:485nm/Em:513nm measurements taken automatically every 10 min. To determine the SOS response during exposure to mitomycin C, cells were grown to early exponential phase growth as described in ‘*Growth conditions.*’ Cells from 1 mL aliquots of each strain were pelleted by centrifugation for 1 min at 13,000 x*g*, and supernatants were removed. Cells were resuspended in 1 mL of LB with the appropriate concentration of antibiotic. One-hundred μL of these cell suspensions were aliquoted into a black-walled, flat bottom 96-well plate (Corning, Corning, NY, United States; Cat #: 3916) and incubated overnight in a Biotek Synergy H1 plate reader, with OD_600_ measurements and Ex:485nm/Em:513nm measurements taken automatically every 10 min.

### Deep Sequencing

Genomic DNA was prepped from overnight cultures prepared from frozen stocks of populations from every even round of selection using the Wizard Genomic DNA Purification Kit (Promega, Madison, WI, United States). DNA samples were submitted to the Department of Energy Joint Genome Institute (Berkeley, CA, United States) for sequencing and analysis. DNA was randomly sheared into ∼500 bp fragments and the resulting fragments were used to create an Illumina library. This library was sequenced on Illumina HiSeq generating 100bp paired end reads. Reads were aligned to the reference genome using BWA (Li and Durbin, 2009), downsampled to an average depth of 250 fold coverage with picard^3^ and putative mutations and small indels were called using callvariants. sh from BBMap.^4^

Sequencing results are reported in their entirety in Supplementary Data 1 a. Hnd 2. However, for analysis of numbers and types of mutations in each population (and consequently for generating Figures 6, 9, 12, and Tables 1, 2 and Supplementary Table S3), mutations in genes with high enough homology to have suspected mismapping of reads (i.e., *rrs*, *rrl*, *rrn*, *rhs*, and *ins* genes) were not considered due to increased likelihood of a false-positive mutation call. In addition, mutations with inconsistent frequency calls (ex: jumping from 0, to 100, to 0% allele frequency) were also not used. All mutations removed from consideration are also listed in Supplementary Data 1 and 2.

**TABLE 1:** Number and types of mutations in evolved populations after 100 cycles of selection.

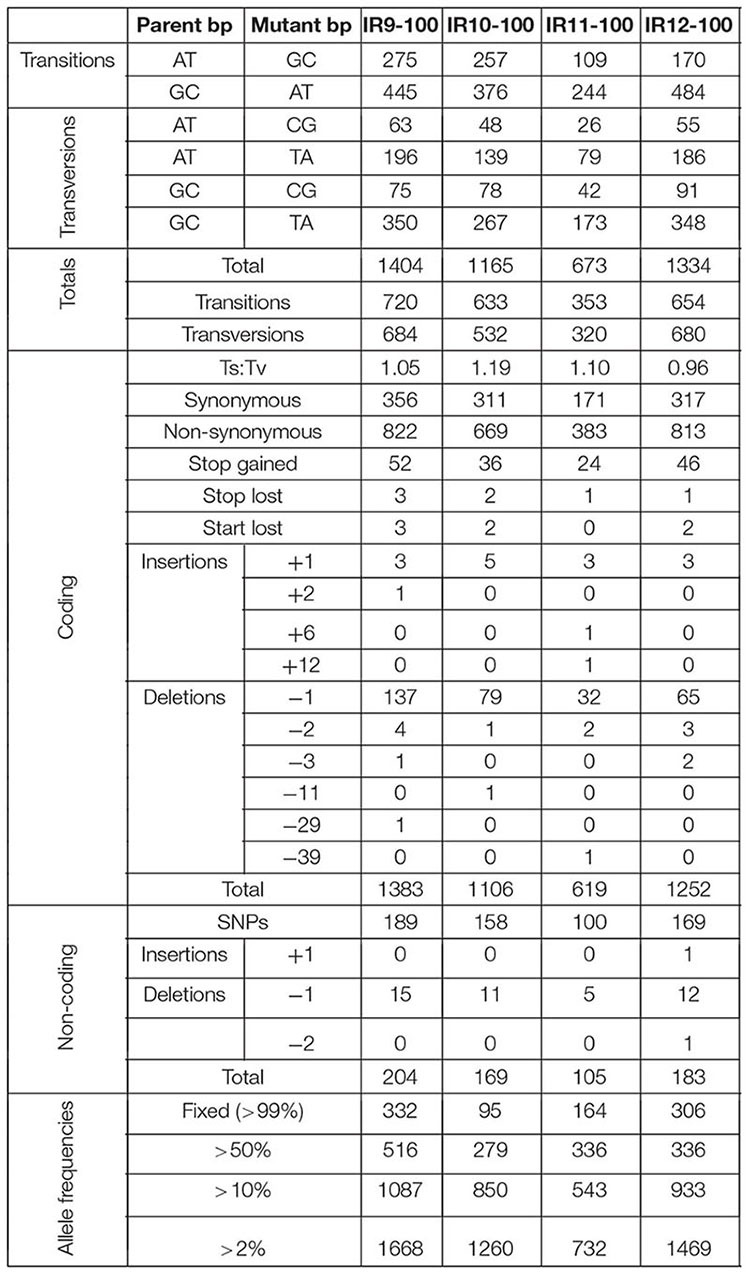

**TABLE 2:** Genetic parallelism indicates candidate driver mutations in each *E. coli* lineage.

**Function**	**Gene**	**IR9**	**IR10**	**IR11**	**IR12**
ATP synthase F1 complex subunit A	*atpA*	**F19L**	-	-	**G199D** R101L
Regulator of BluR - Biofilm formation and acid resistance	*bluF*	V268L	-	**E368A**	**R186W S318P**
Cu2 + chaperone	*copA*	**V270F** Q660R	-	**T525A**	**A812V**
RecA regulator	*dinI*	**R28H**	W71*	**S26 fs**	-
Heat-shock chaperone protein	*djlC*	-	**R312W**	-	**A202V**
Aldohexuronate transporter	*exuT*	**S174R**	-	**F400Y**	-
Fatty-acid biosynthesis regulator	*fabR*	**V3E** F48L	**A38E**	-	-
Formate dehydrogenase subunit	*fdoG*	**C53F**	**S160C**	-	D66A
Heat-shock chaperone protein	*fkpA*	**D196 fs**	-	**A260T**	-
Membrane-associated protein	*glcF*	-	-	**N186K**	**R165L**
Glycogen biosynthesis	*glgB*	**N146S**	F18L	-	**W298L**
Glutaredoxin 3	*grxC*	-	-	**Q61E**	**L22Q**
Glutatione transporter	*gsiA*	**P572T** R176Q	-	-	**E194Q**
Catalase II	*katE*	**L198I**	-	**H449P** A30fs	D643Y
Potassium dependent sensory histidine kinase	*kdpD*	**D723 fs** A364T	P8A A657T	-	**L291 fs**
2-keto-3-deoxy-D-gluconate dehydrogenase	*kduD*	H179R	**K11N**	**E82V**	-
Putative efflux pump subunit	*mdlB*	**G550 fs Q473 fs**	-	-	E395*
Peptidoglycan synthesis	*mrdB*	-	**R363M**	-	**K232T**
Nitrate reduactase A subunit	*narH*	-	**D398Y R413H**	-	**D488Y**
NADH:quinone oxidoreductase II	*ndh*	D280G	-	**A393 fs**	**A15 fs**
Endo III	*nth*	L371 fs	C203Y I79L V36 fs	**T43 fs**	**K85N**
Chaperone for PaoABC oxidoreductase	*paoD*	-	**E171K**	-	**K14N**
Paraquat inducible protein	*pqiA*	**A367T** P176S G216R	-	-	**A21V W177L** D198Y W197*
PEP phosphotransferase	*ptsP*	-	**R119 fs**	K11R R526C	**D641G**
RecBCD subunit	*recD*	**A90E** G362D	N124D Q463*	**A550E**	**S92I**
ssDNA exonuclease	*recJ*	*578L M360I	G502D	-	**F426L** T454N
Cohesin-like protein	*recN*	**K429Q**	R415L	**R102P**	**A361T** A512T
RNAP beta-subunit	*rpoB*	**S72N** D842E	V630E K1200E	**P535L S574F**	**T600I** V146F R557C Y872C
Putative fimbral usher protein	*sfmD*	**D672Y**	W73*	-	**A241T**
Tyrosine t-RNA ligase	*tyrS*	**A2S**	-	-	**A109T**
Putative LPS synthesis	*waaU*	**A172P**	-	-	**H323Y** I30V
Putative colanic acid synthesis	*wcaL*	**G312 fs**	-	-	**R255C G262A** T23N
Putative transporter	*yaaU*	-	-	**I384T**	**F265Y**
Putative transporter	*ybjL*	**A512 fs**	V45A	M67T	**L476F**
Putative transporter	*yebQ*	**R140 fs** L401*	-	-	**I246 fs**
Putative autotransporter	*yfaL*	**R630 fs L770F** G326V	G203 fs	**P1177R**	G800C
Putative transporter	*yihN*	**W103C**	-	**V256A**	-
Inner-membrane protein	*yrfF*	**A522V** S173A	-	**R29C**	-

*Proteins listed are encoded by genes that have i) mutations that were non-synonymous, and ii) fixed in at least two populations at round 100. The bolded variants are fixed in the indicated population. The non-bold variants are those that are not fixed but are present at least at 2% frequency in the indicated population at round 100 of selection.*

### Oxford Nanopore Sequencing

Approximately 1 × 10^9^ early exponential phase (OD_600_: 0.2) cells of evolved isolate IR9-100-2 were pelleted and delivered to the University of Wisconsin – Madison Biotechnology Center for genomic DNA isolation and preparation. DNA was sequencing using an Oxford Nanopore (Oxford Nanopore Technologies; Oxford, United Kingdom) platform. Reads from the resulting. fastq file were assembled using DNASTAR SeqMan Pro software. Contigs longer than 10 kb were then searched for homology near the deleted or duplicated regions in IR9-100-2.

### Cell Lysis for Organic and Aqueous Compound Extraction

Appropriate strains were cultured and spread plated as according to ‘*Growth conditions*’ and ‘*Serial dilutions.*’ To facilitate porphyrin accumulation in IR9-100-2, all plates were incubated at 37°C for 72 h, and then at room temperature for 1 week. Isolated colonies were collected from plates using a sterile wooden stick and were resuspended in 1 mL of 1X PBS. Enough colonies were collected to total ∼ 100 μL of cells. Cells were pelleted by centrifugation at 13,000 x*g* for 1 min, and supernatants were removed. Cells were resuspended in 300 μL dH_2_O, 400 μL MeOH, and 100 μL CHCl_3_. Cells were mixed by vortexing vigorously for 20 s, and then centrifuged for 5 min at 13,000 xg to separate aqueous and organic layers. The aqueous layer was removed and stored in a new 1.5 mL Eppendorf tube. Five-hundred μL of MeOH was added to the protein/organic layer, and the sample was centrifuged at 13,000 x*g* for 1 min to pellet protein.

### Spectral Analysis of Aqueous Extract

One-hundred μL of aqueous extracts were pipetted into a flat bottom, black walled 96 well plate (Corning, Corning, NY, United States; Cat #: 3916). Using a Biotek Synergy H1 plate reader (Biotek, Winooski, VT, United States), an absorbance scan was performed from 300 nm to 700 nm. Given the absorption maximum at 395 nm, next an emission scan was performed from 400 to 700 nm.

### TMT-Labeled Mass Spectrometry

Cells were prepared and irradiated, and tandem mass tag (TMT) mass spectroscopy were performed as previously described (Bruckbauer et al., 2020). Briefly, 10μg (varying volumes) of purified protein from each of the 10 samples (5 treated with 1,000 Gy, 5 untreated) was diluted to 4M urea with 50mM TEAB and treated with 2mM dithiothreitol for 30 min at 50°C, 5mM iodoacetamide for 30 min at room temperature in darkness, and then 2mM dithiothreitol for 5 min at room temperature. Samples were diluted further to 1M urea with TEAB, and 0.05 μg of Trypsin and Lys-C proteases were each added (final protease mass:protein mass of 1:100). Samples were incubated overnight at 37°C, for 15 h total.

Digestions were stopped with addition of neat formic acid to 1.0%, subjected to solid phase cleanup and dried down to completion using a vacuum centrifuge. Dried samples were resolubilized into 50mM TEAB. To each tube, 40 μg of each of the 10 tandem mass tag chemical adducts were added as previously described. After reaction quenching, samples were immediately combined and purified/concentrated using a Waters 1cc C18 Sep-pak solid phase chromatographic column (Waters Corporation; Milford, MA, United States), according to manufacturer’s protocol, then dried to completion with a vacuum centrifuge. Samples were resolubilized into 300 μL 0.1% formic acid for high-pH tip-based fractionation. Fractionation was carried out using a Pierce high pH reversed phase peptide fractionation spin column kit (Pierce Corporation; Junction City, OR, United States), according to manufacturer’s protocol. These fractions were dried down to completion using a vacuum centrifuge and resolubilized into 10 μL 0.1% formic acid for injection onto a Thermo Fisher Orbitrap Lumos mass spectrometer (Thermo Fisher; Waltham, MA, United States). For liquid chromatographic conditions, stationary phase was C18 and flow rate was 275 nL/min. Three μL of each fraction was injected for analysis. Mobile phases A and B were 0.2% formic acid in water and 0.2% formic acid in 70% acetonitrile, respectively. For separation and elution, a 60 min gradient to 55% buffer B was used. HCD fragmentation was used to obtain high resolution MS2 spectra for TMT mass tag quantification.

Data was analyzed using the Sequest algorithm within Proteome Discoverer (PD) (Thermo Fisher; Waltham, MA, United States) as previously described. For TMT quantification, tag abundances were normalized to the total tag/peptide amount per channel. An average of normalized abundance for the 1kGy channels over the 0Gy channels was used to determine peptide level fold change, and two-tailed t-testing was used to calculate a *p*-value per peptide. Adjusted p values were then calculated using Benjamini-Hochberg correction.

## Results

### Ionizing Radiation Resistance Has Continued to Increase Since Cycle 50 of Selection

Using our previously published selection protocol (Bruckbauer et al., 2019b), we continued experimental evolution of IR resistance in *Escherichia coli* for 100 total cycles of selection. The lineages are denoted IR9, IR10, IR11, and IR12. Each has a further numerical designation indicating the given cycle of selection for each population (ex: IR9-100 is a population from lineage IR9 at cycle 100 of selection). Briefly, at each selection cycle, cultures of each population were grown to early exponential phase in LB rich medium, cooled on ice to stop growth, and then washed in cold phosphate-buffered saline (PBS) to remove any components of the growth media which may promote metabolism or ameliorate reactive oxygen species (ROS) generated by IR. Each culture was then exposed to sufficient IR to kill 99% of the population. Following irradiation, a portion of the population was assayed for survival rate while the rest was outgrown overnight in rich medium. This overnight culture was then stored at −80°C as the population used for the next round of selection.

We have noted previously (Bruckbauer et al., 2019b) that all strains tested, including IR-resistant isolates from previous trials using ^60^Co and *Deinococcus radiodurans* itself, are more sensitive to the high dose rate of the high energy electron beam irradiation provided by the Linac than they are to the ^60^Co irradiation. This can readily be seen in the reduced survival seen here for *Deinococcus radiodurans* at 5,000 Gy relative to that reported previously (Cox and Battista, 2005; Daly, 2009; Slade and Radman, 2011).

**FIGURE 1:**
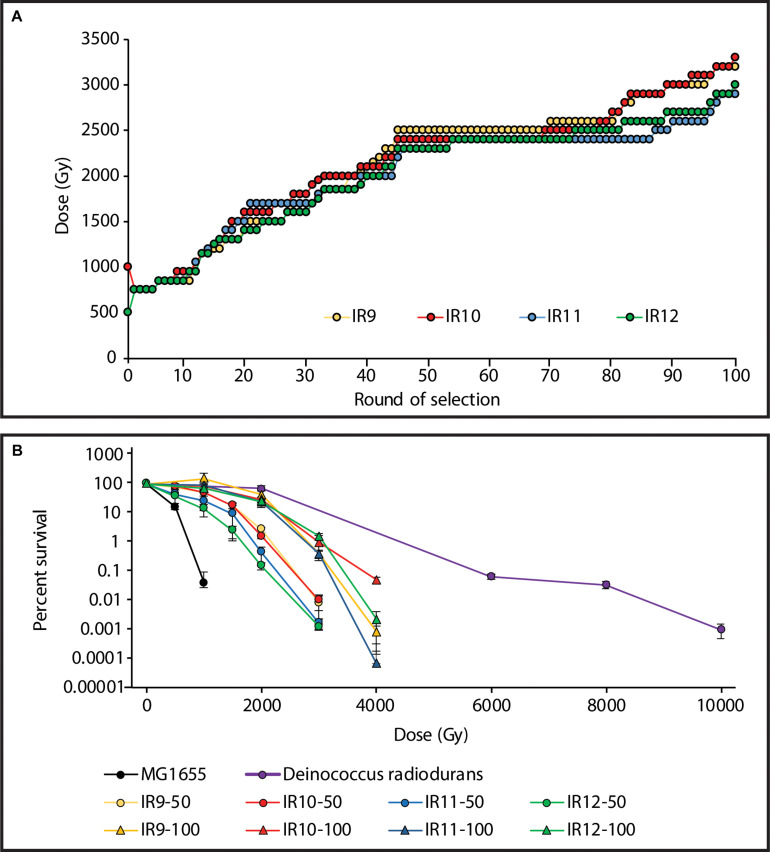
Experimentally evolved ionizing radiation resistance has continued to increase over 100 cycles of selection. **(A)** Dose required to kill 99% of each population has increased. Each data point indicates the dose of IR that each population was given prior to being outgrown overnight and stored at –80°C. The percent survival was estimated from a single replicate at each round of selection. **(B)** Survival curves of evolved populations compared to previously evolved *Escherichia coli* isolates and *Deinococcus radiodurans*. MG1655 is the Founder strain used to begin the evolution experiment. IR’X’-50 and IR’X’-100 are populations of the indicated lineages after 50 or 100 cycles of selection with IR. Early exponential phase cultures of the indicated strains were exposed to electron beam IR as described in the Materials and Methods. Error bars represent the standard deviation of CFU/mL calculations from a single experiment performed in biological triplicate. Survival data for MG1655, populations after 50 cycles of selection, and *D. radiodurans* strain R1 are as previously reported (Bruckbauer et al., 2019b).

Using the Linac, the dose required to kill 99% of each population increased from ∼750 Gy after the first cycle, to 2,300-2,500 Gy after 50 cycles of selection (Bruckbauer et al., 2019b). After 100 total cycles of selection, this further increased to 2,900-3,200 Gy (Figure 1A). The four populations at cycle 100 of selection (IR9-100, IR10-100, IR11-100, and IR12-100) all exhibited approximately 100-fold increased survival after a dose of 3,000 Gy compared to the same lineages at round 50 (Figure 1B). Notably, these populations have begun to develop a shoulder of IR resistance comparable to the highly radioresistant bacterium *Deinococcus radiodurans*, with little loss in viability at a dose of 2,000 Gy.

### Evolved Isolates Exhibit Fitness Trade-Offs

**FIGURE 2:**
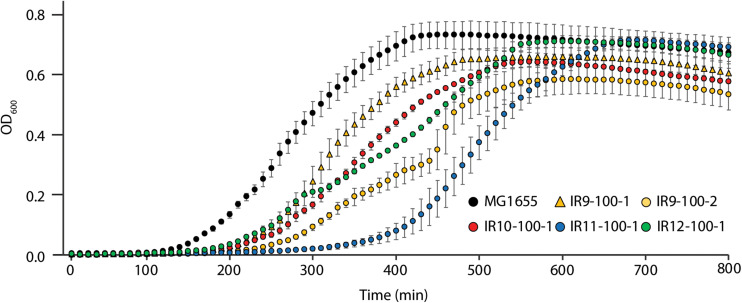
Evolved isolates exhibit growth defects in the absence of IR selection. MG1655 is the Founder strain used to begin the evolution experiment. Strains named IR’X’-100-1 are isolates from the indicated mixed population at 100 cycles of selection. IR9-100-2 is a second isolate from population IR9-100. Cultures of indicated strains were grown in LB medium overnight and then to early exponential phase as described in the section “Materials and Methods.” All cultures were incubated at 37°C during growth. Early exponential phase cultures were diluted 1:100 in the appropriate medium, and then incubated overnight in a Biotek Synergy 2 plate reader, with OD_600_ measurements taken automatically every 10 min. This experiment is representative of two independent experiments performed in biological triplicate; error bars represent the standard deviation of the OD_600_ measurements of a single biological triplicate.

After 50 cycles of selection, isolates from each experimentally evolved population had no noticeable growth defect in rich medium (Bruckbauer et al., 2019b). However, isolates from each population after 100 cycles of selection exhibited a growth deficiency in rich medium without IR selection. This was reflected in part by a lag in recovery from stationary phase and in some cases by impaired growth rates (Figure 2). This apparent decrease in fitness was variable within populations, as demonstrated by differences in growth curves between two isolates from IR9-100 (IR9-100-1 and IR9-100-2).

**FIGURE 3:**
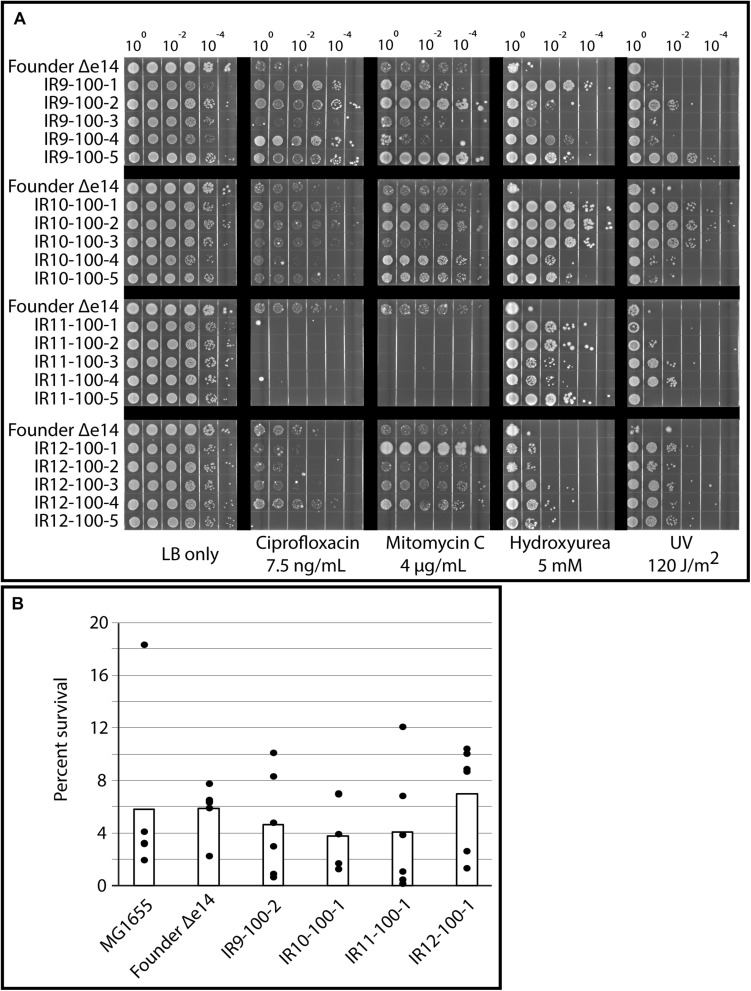
Evolved isolates do not exhibit broad resistance to DNA damage. **(A)** Evolved isolates have variable resistance to DNA damaging compounds and UV. Five isolates from each population and the Founder strain (lacking the e14 prophage, which is lost early in the evolution experiment Bruckbauer et al., 2019b) were grown in LB medium overnight and then to early exponential phase as described in the section “Materials and Methods.” Cultures were serial diluted in 1X PBS, and then 10 μL of each dilution was spot plated onto LB agar with the indicated DNA damaging agent. Once spots dried, the plates were then incubated overnight before imaging. For UV exposure, 10 μL of each dilution was spot plated onto LB agar and exposed to the appropriate dose of UV once spots dried. Results shown are representative of two independent experiments performed. **(B)** Evolved isolates are not significantly more desiccation tolerant than the Founder strain. A single isolate from each population after 100 cycles of selection, the Founder strain of *E. coli* (MG1655), and the Founder strain lacking the e14 prophage and placed in a desiccator for 1 week and assayed for survival as described in the Materials and Methods. Three independent experiments in biological triplicate were performed; black circles indicate the percent survival of each replicate, and bars indicate the average of each triplicate. There is no significant difference between survival of each strain, as calculated by a Student’s two-tailed *T*-test (*p*-value > 0.1).

Growth deficiencies were accompanied by clear specialization to survive damage induced by IR exposure. As previously observed in isolates from round 50 of selection (Bruckbauer et al., 2019b), isolates from round 100 of selection exhibited variable levels of resistance to UV-C irradiation and a variety of DNA-damaging compounds (Figure 3A). Desiccation stress leads to severe DNA damage similar to that caused by IR exposure (Dose et al., 1992), and IR resistance is often linked to desiccation tolerant organisms (Rainey et al., 2005). As such, we assayed for desiccation tolerance in our evolved *E*. *coli* populations. However, isolates from each population exhibited no increase in desiccation resistance compared to the Founder strain in our assay (Figure 3B). The lack of desiccation resistance suggests that these evolved populations are not exhibiting convergent evolution toward the highly desiccation and IR-resistant phenotype of *D. radiodurans*, and is in fact developing highly selective resistance to IR. In accordance with this hypothesis, isolates from round 100 of selection do not have significantly altered ratios of intracellular Mn and Fe (Supplementary Figure S1), which has been hypothesized to be an indicator of IR resistance in nature (Daly et al., 2004, 2010; Sharma et al., 2017).

### Evolved Isolates Exhibit Differential Responses to ROS Stress and DNA Damage

**FIGURE 4:**
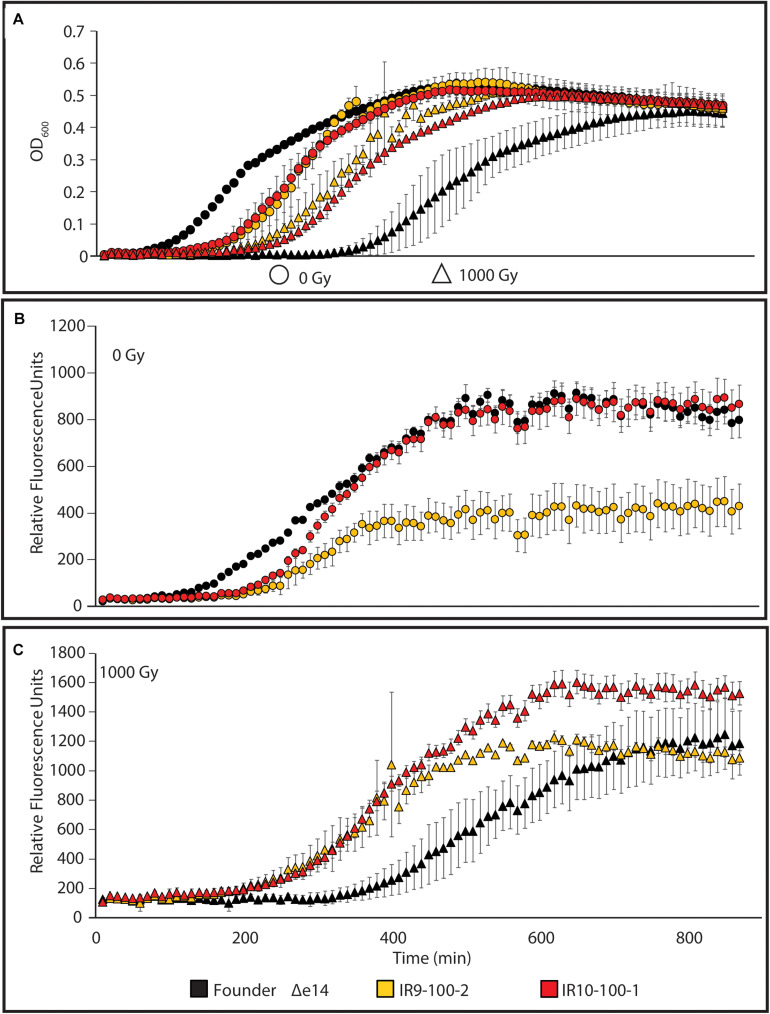
The SOS response to DNA damage in evolved isolates. **(A)** Growth of evolved isolates post-IR exposure. The evolved isolates are from two separate lineages after 100 cycles of selection. Each exhibits a growth defect compared to Founder Δe14 without IR selection. After irradiation with 1,000 Gy, evolved isolates recover more quickly than Founder Δe14. **(B)** P*_*recN*_* driven expression of GFP at 0 Gy or **(C)** 1,000 Gy. The relative SOS response of each strain was assayed using the SOS-controlled promoter of the *recN* gene driving expression of GFP. GFP expression was normalized to the OD_600_ values indicated in panel **(A)**. Cultures of indicated strains were grown in LB medium overnight and then to early exponential phase for irradiation as described in the section “Materials and Methods.” After irradiation, mock-treated and irradiated cultures were diluted 1:100 in the appropriate medium, and then incubated overnight in a Biotek Synergy 2 plate reader, with OD_600_ measurements and Ex:485nm/Em:513nm measurements taken automatically every 10 min. This experiment is representative of two independent experiments performed in biological triplicate; error bars represent the standard deviation of the OD_600_ [panel **A**] or fluorescence normalized to OD_600_ measurements [panels **(B,C)**] of a single biological triplicate.

We sought to determine if such variable survival to DNA damaging agents is reflected in a differential SOS response (as a proxy for DNA damage detection). Therefore, we assayed the response in an isolate from IR9-100 (IR9-100-2) and an isolate from IR10-100 (IR10-100-1), with or without exposure to 1,000 Gy of IR. To assay the SOS response, we utilized a previously characterized fusion of the promoter for the gene encoding the DNA-damage responsive protein RecN with GFP and monitored GFP expression throughout growth after IR exposure (Figure 4). The level of P*_*recN*_*-driven GFP expression is normalized to the OD600 of each strain grown without irradiation or after irradiation with 1,000 Gy.

As seen in Figure 2, the two evolved isolates exhibited a lag in growth in the absence of irradiation but growth recovered more quickly for the isolates than the Founder strain after exposure to 1,000 Gy (Figure 4A). This probably reflects differences in cell survival after irradiation. In the absence of exogenous DNA damage, IR10-100-1 displays an SOS response similar to wild-type cells (Figure 4B). Interestingly, IR9-100-2 exhibits approximately a 2-fold lower maximum SOS signal compared to Founder Δe14 (the Founder strain lacking the e14 prophage, which was lost early in the evolution experiment in three of the four populations) and IR10-100-1 in the absence of IR (Figure 4B). After exposure to IR, each strain exhibits SOS induction similar to Founder Δe14, with a small but significant increase in maximal SOS induction in IR10-100-1 (Figure 4C). The level of IR-induced SOS in Founder Δe14 noted here is lower than that observed by induction from mitomycin C exposure during growth (Supplementary Figure S2). Overall, adaptation to IR exposure has moderately altered the SOS response to DNA damage in a manner that varies from one isolate to another.

**FIGURE 5:**
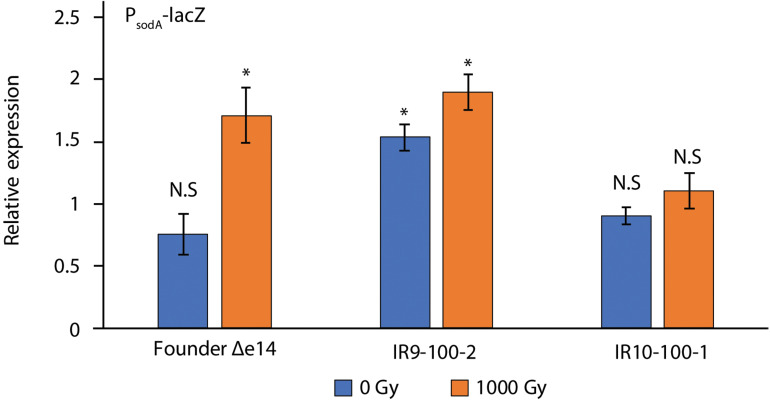
β-galactosidase activity of P*sodA*-*lacZ* fusions in Founder Δe14 and evolved isolates. To assay the cellular response to ROS stress, we utilized a fusion of the promoter of superoxide dismutase A (*sodA*) with the *lacZ* gene (Giel et al., 2006). β-galactosidase activity was assayed in exponential phase cultures incubated for 1 h in LB after mock-treatment or exposure to 1,000 Gy IR, as described in the Materials and Methods. The outgrowth step was included to allow a cellular response after irradiation. β-galactosidase activity was normalized to values for unirradiated Founder Δe14 culture prior to the outgrowth step. Data represent the results of two independent experiments carried out in biological duplicate. Statistical significance was determined using a two-tailed Student’s *t*-test. *P*-values are indicated using the ‘*’ (*p* < 0.01) and ‘N.S.’ (*p* > 0.01).

Although each compound tested in Figure 3A causes DNA damage, ciprofloxacin, UV-C, and mitomycin C damage DNA directly. UV-C and hydoxyurea may also generate damage via an ROS-mediated mechanism (Sakano et al., 2001; Santos et al., 2013). Therefore, variable resistance to these compounds may be a product of an altered response to ROS stress. To assay the stress response to ROS, we utilized the promoter for the gene encoding superoxide dismutase A (*sodA*) to drive expression of β-galactosidase (LacZ) (Figure 5). After a dose of 1,000 Gy, P*_*sodA*_* activity in Founder Δe14 increases by 2-fold. IR9-100-2 and IR10-100-1 exhibit little to no IR-dependent increase in P*_*sodA*_* activity. However, the basal P*_*sodA*_* activity in IR9-100-2 is 2-fold higher than that of Founder Δe14. Therefore, both IR9-100-2 and IR10-100-1 exhibit modest alterations in the ROS stress response, although the modified response appears as constitutive activation (IR9-100-2) or reduced sensitivity to induction (IR10-100-1).

### The Mutational Landscape of Evolved Populations After 100 Cycles of Selection

**FIGURE 6:**
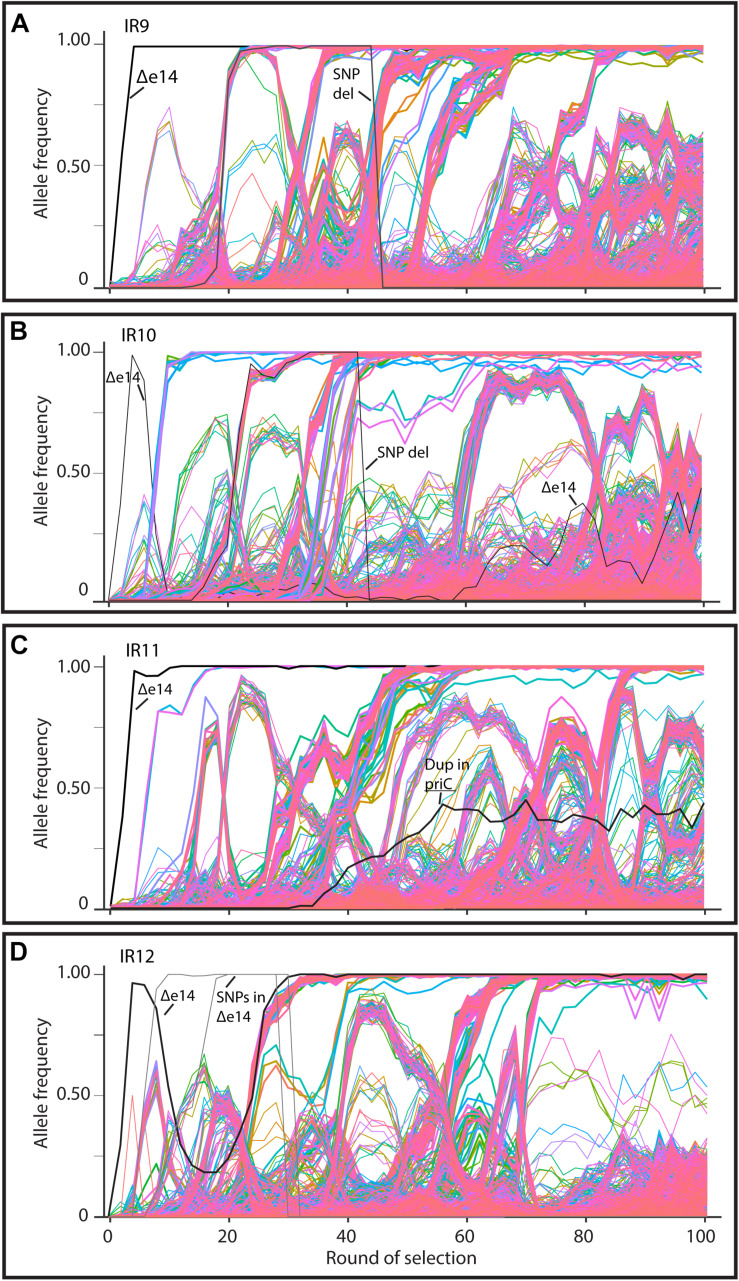
Allele frequencies of evolving populations throughout 100 cycles of selection. Each line represents a mutation in lineages **(A)** IR9, **(B)** IR10, **(C)** IR11, or **(D)** IR12. As mutations rise in frequency, at least one is considered to be a ‘driving’ mutation which enhances fitness of the clone in which it appeared. Therefore, in each cluster of mutations, the ‘drivers’ are accompanied by hitchhiking mutations which are likely neutral, or perhaps deleterious, in their effect. Once mutations reach 100% frequency, these mutations are fixed in the population and all further mutations will occur in this genetic background. A strongly beneficial mutation will lead to a quick rise in frequency of it and any associated hitchhiking mutations; such groups of mutations therefore ‘sweep’ through the population. Selective sweeps come at the expense of other competing lineages within a population, so as sweeps move through a population, other less-fit sub-lineages are driven extinct. A dramatic, early example of this phenomenon occurs in lineage IR10, where the sub-lineage that lost the e14 prophage was driven extinct by a second sweep that had not lost the prophage **(B)**. Thicker lines represent mutations that fix by round 100 of selection. The black lines in each graph represents loss of the e14 prophage, mutations lost in a deletion event, or a small duplication event within the indicated gene. Genomic DNA was prepared from freezer stocks of whole populations from each even round of selection to round 100. Samples were submitted to the Department of Energy Joint Genome Institute for resequencing using an Illumina Hi-seq. On average, 200X coverage was achieved which allows detection of mutations present at as low as 2% frequency.

As in the previous report (Bruckbauer et al., 2019b), we have subjected all four evolving populations to deep-sequencing after every even round of selection to round 100. Reads from each whole-population sample are mapped to the Founder reference genome, at which point mutations can be detected and their frequency quantified due to the proportion of reads containing the polymorphism at that location. We can thus quantify the frequency each mutation present at a minimum of 2% frequency within each evolving lineage. Stitching together the static views of allele frequencies at each selection cycle reveals the dynamics of polymorphism abundance over time, and by extension, the prevalence of driving mutations and clonal interference in each evolving lineage (Figure 6). Figure 6 depicts each mutation as a single line, with the frequency of this mutation changing over time if the clone containing the mutation is more fit (increasing) or less fit (decreasing) than the other competing clones at the given cycle of selection. As clones compete with one another in the evolving lineages, the rate at which mutations increase in frequency decreases due to clonal interference where potentially beneficial mutations are prevented from fixing in the population due to the other competing clones.

Despite significant clonal interference, each population has had numerous selective sweeps (as defined by groups of mutations progressing together to reach 100% frequency at the same round of selection) throughout the evolution process. Lineage IR9 has had four distinct sweeps since round 50 of selection. Lineages IR10, IR11 and IR12 have had one, three, and five sweeps since round 50, respectively.

Each population is highly mutagenized by round 100 of selection. Three of the four populations (IR9, IR10, and IR12) have over 1,000 single nucleotide polymorphisms (SNPs). IR11 has far fewer SNPs, at 632 (Table 1). In all four populations, most polymorphisms affect coding regions (approximately 87% of all polymorphisms in each population). As previously observed at cycle 50 of selection (Bruckbauer et al., 2019b), transition mutations make up approximately 57% of all mutations in each of the populations. Small insertions and deletions (± 1 to 39 bp) are present, where -1 deletions are the most common mutation of this type in all four populations.

Larger genomic deletions are occurring at increasing frequency. In our previous study, the only large deletion noted was excision of the e14 prophage. This excision event occurred in all four populations at the beginning of the evolution experiment. However, in lineage IR10 the subpopulation that lost e14 was outcompeted and the presence of this prophage was maintained by cycle 50 of selection (Bruckbauer et al., 2019b). Now at cycle 100, population IR10-100 contains a subpopulation that has lost the e14 prophage and has reached approximately 50% frequency. If the loss of e14 is eventually fixed in lineage IR10, this prophage deletion will be the only mutation to have occurred in all 4 lineages. A contribution of e14 deletion to IR resistance has been documented previously (Byrne et al., 2014).

**FIGURE 7:**
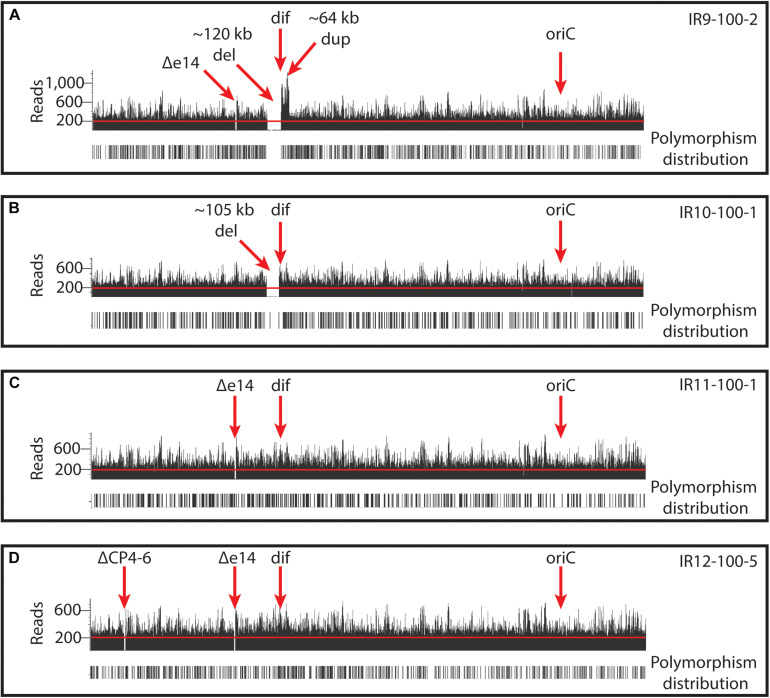
Deep-sequencing read depths in each evolved population reveal large genomic deletions and duplications. Read depths are illustrated for representative isolates from **(A)** IR9-100, **(B)** IR10-100, **(C)** IR11-100, and **(D)** IR12-100. Illumina read depths across the genome of representative evolved isolates. The origin of replication (*oriC*) and *dif* sequence (where replication terminates) are indicated. Read depths reveal deletions of prophages (e14 and CP4-6) and ∼100kb deletions in IR9 and IR10. In addition, IR9 contains an ∼65 kb duplication adjacent to the deletion. Black bars below read depths indicate the location of a polymorphism. Each graph of reads depths is of sequencing of an isolate which is representative of other sequenced isolates from the same population. Graphs were generated by aligning assembled reads (.bam files) to the *E. coli* MG1655 U00096.3 (NCBI GenBank accession number) using Golden Helix GenomeBrowse 3.0.0 software.

**FIGURE 8:**
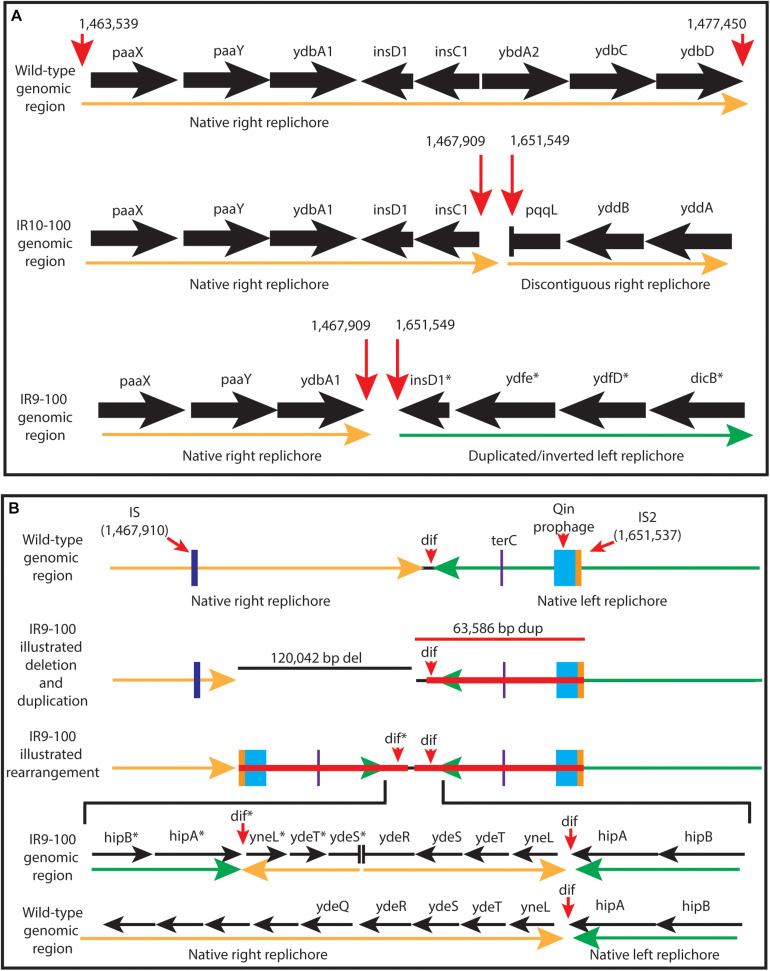
Characterization of the approximately 100 kb deletion in IR9 and IR10. **(A)** A cartoon depiction of the deletion in IR9 and IR10. In both lineages, the deletion begins near an insertion element, but have differing beginning and end locations (∼120 kb deletion in IR9, and ∼105 kb deletion in IR10). In IR10, the deletion truncates the *pqqL* gene. In IR9, the deletion truncates the *ydeR* gene, but the deleted area is replaced by a duplicated and inverted genome sequence [see panel **(B)**]. **(B)** Cartoon depiction of the IR9 duplication. The duplication replaces the insertion element on the right replichore (IS) with that of the left replichore (IS2). Approximately 80 genes are duplicated, with major duplicated elements including the Qin prophage, and the *terC* and *dif* genetic elements. The overall structure of the duplicated region was confirmed via Oxford Nanopore sequencing, as described in the Materials and Methods. Locations of Oxford Nanopore reads are shown in Supplementary Figure S3.

New analysis of Illumina sequencing read depths has revealed a massive, single deletion of approximately 100 kbp in IR9 and IR10 at the same approximate location near the replication terminus in both lineages (Figure 7). The deletion first appeared at round 28 (IR9) and 36 (IR10) and fixed by round 36 and 40, respectively. Nearly 100 predicted open reading frames are deleted in each lineage (Supplementary Table S2). While the start position of each deletion is similar (occurring before or after the insertion element inserted within the *ydbA* gene, respectively, in IR9 and IR10) the deletion extends approximately 10 kb further in IR9 (into the *ydeR* gene; 120,050 bp deleted) compared to IR10 (into the *pqqL* gene; 105,697 bp deleted) (Figure 8A). The genes deleted in each lineage are listed in Supplementary Table S2.

Interestingly, the buildup of Illumina sequencing reads adjacent to the deletion in IR9 indicates that the 65 kbp immediately downstream of the *ydeR* gene is duplicated and inverted in lineage IR9 (Figure 8B). This duplication appeared alongside the 120 kbp deletion at round 28 of selection. Within the duplicated region are the genetic elements *dif* and *terC*, both important for proper termination of genome replication, as well as the majority of the cryptic Qin prophage. Seventy-nine genes are duplicated in this 65 kbp region (Supplementary Table S2). We have confirmed the presence and overall structure of this rearrangement using Oxford nanopore sequencing (locations of Oxford nanopore reads are depicted in Supplementary Figure S3). However, approximately 1,800 bp (containing the region from the duplicated *ydeS* through *hipA* genes) appears heavily mutated. Attempts to sequence this region using conventional Sanger methodology have thus far proven unsuccessful. Therefore, we likely do not yet have a complete understanding of the nature of this duplicated region. Given the presence of insertion elements at the 5′ end of the deletion in both IR9 and IR10, and at the 3′ end of the duplicated region in IR9, we hypothesize that these structural rearrangements are insertion element-mediated (Figure 8B). The full implications of these substantial structural changes on genome stability and maintenance remain to be explored.

### Clonal Interference Is Pervasive in All Four Evolving Lineages

**FIGURE 9:**
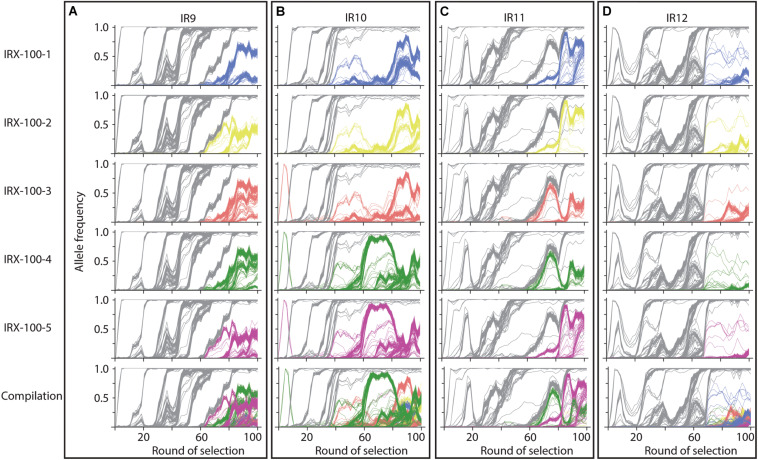
Evolutionary histories of sequenced isolates from populations at round 100 of selection. At round 100 of selection, there are two competing subpopulations present at high frequencies in lineages **(A)** IR9, **(B)** IR10, and **(C)** IR11. There are no major subpopulations in **(D)** IR12. Each row represents a sequenced isolate in the indicated lineage, which is indicated by each column. Alleles in gray are those present in all isolates; colored alleles appear in the given isolate but are not fixed within the population. The bottom row consists of the combined allele frequency graphs of each isolate to illustrate the competition between underlying sub-populations.

Significant clonal interference has delayed the progress of sweeps in each population. The evolutionary history of the 5 sequenced isolates from each population at round 100 of selection reveals that lineage IR9, IR10, and IR11 each have at least two distinct sub-populations of at least 25% frequency by round 100 of selection (Figure 9). These competing subpopulations have been maintained for approximately 40 cycles of selection in IR9 and IR11, and approximately 60 cycles in IR10. No such competition has become apparent in IR12. It is unclear if this coexistence represents a developing cooperativity between clonal lineages, or the fitness increase necessary to allow a sub-population to sweep to fixation is becoming more difficult due to diminishing returns epistasis.

**FIGURE 10:**
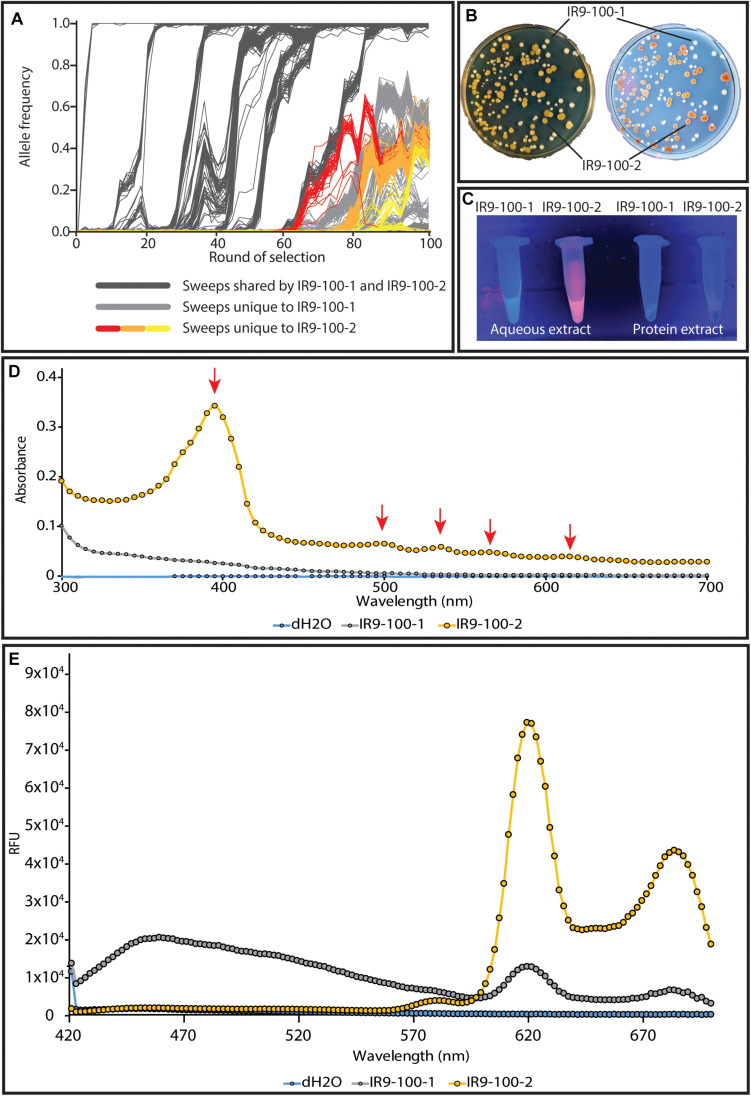
The two distinct subpopulations of lineage IR9. **(A)** The two subpopulations are highlighted amongst the frequencies of all mutations in evolving lineage IR9. **(B)** A representative plate of IR9-100 demonstrates the divergent colony phenotypes of each subpopulation. The plate was incubated for 72 h at 37°C to allow the yellow-brown coloration to fully develop. **(B)** Yellow-brown colonies fluoresce when exposed to UV light. **(C)** Aqueous extracts of isolates from IR9-100 suggest porphyrin accumulation in the yellow-brown isolate IR9-100-2. Extracts were prepared as described in the section “Materials and Methods.” Fluorescence of the aqueous extract when exposed to UV light suggested that the causative compound was extracted in the aqueous layer. **(D)** An absorbance spectrum of the aqueous extract was performed to determine an optimal excitation wavelength of the compound. **(E)** With a maximum excitation wavelength of 395 nm, an emission spectrum was performed. Absorbance and emission spectra were performed on a Biotek Synergy H1 as described in the section “Materials and Methods.”

We have begun to investigate the nature of the relationship between the two major subpopulations of lineage IR9. Prior to any available sequencing data, these populations were distinct from one another due to the yellow-brown colony phenotype of one of these populations (Figure 10A). IR9-100-2 is a member of this yellow-brown subpopulation (Figure 10B). Much characterization remains to be done to determine the genetic drivers of these two subpopulations, the evolutionary relationship between the two (ex: competitive vs. cooperative) and the genetic origin of the yellow-brown phenotype. However, preliminary experiments suggest the colony coloration is caused by accumulation of porphyrin species, the metabolic precursors to heme. IR9-100-2 exhibits UV-induced colony fluorescence that is typical of high-level porphyrin (Kazmi and Bukhari, 1978; Tatsumi and Wachi, 2008; Turlin et al., 2014; Figure 10B). Aqueous extract of IR9-100-2 cells additionally exhibit UV-induced fluorescence (Figure 10C), strongly absorbs at a wavelength of approximately 395 nm (Figure 10D), and when excited with this wavelength emits at peaks of 619 and 683 nm (Figure 10E). These spectral properties match those of porphyrin species, particularly coproporphyrin III (Layer et al., 2002; Dietel et al., 2007; Ding et al., 2019). Furthermore, previous studies have demonstrated that porphyrin accumulation can dramatically alter colony coloration, as seen in IR9-100-2 (Cox and Charles, 1973; Kwon et al., 2003).

### Mutations Which Contribute to IR Resistance in IR9-100-2

We set out to define some of the mutations contributing to the IR resistance phenotype, focusing on isolate IR9-100-2. We generated a list of candidate mutations implicated in driving IR resistance in a similar manner to previous studies (Byrne et al., 2014; Bruckbauer et al., 2019b). We focused our attention on genes with a fixed mutation in at least two populations at round 100 of selection (Table 2). These genes are involved in a wide array of pathways, affecting nucleotide metabolism, DNA repair, ROS amelioration, cell wall and membrane metabolism, ATP synthesis, and more. At cycle 100 of selection, the *recD*, *recN*, and *rpoB* genes are the only genes of known function with a mutant allele present at some frequency in all four populations (with *recD* being the only gene with fixed alleles in all four populations). Variants of the proteins encoded by these genes were previously demonstrated to enhance IR resistance (Bruckbauer et al., 2019b), suggesting that genetic parallelism was a prevalent marker for the drivers of evolved IR resistance prior to round 50 of selection. As the populations diverge, genetic parallelism is becoming less reliable as an indicator of phenotypic contributions.

We previously determined that variants of RecD, RecN, and RpoB/C accounted for the majority, but not all, of the IR-resistant phenotype of IR9-50-1, an isolate from IR9 after 50 cycles of selection (Bruckbauer et al., 2019b). IR9-100-2 is a descendant of IR9-50-1 and we sought to identify mutations which have driven the four selective sweeps since cycle 50 (Figure 6).

**FIGURE 11:**
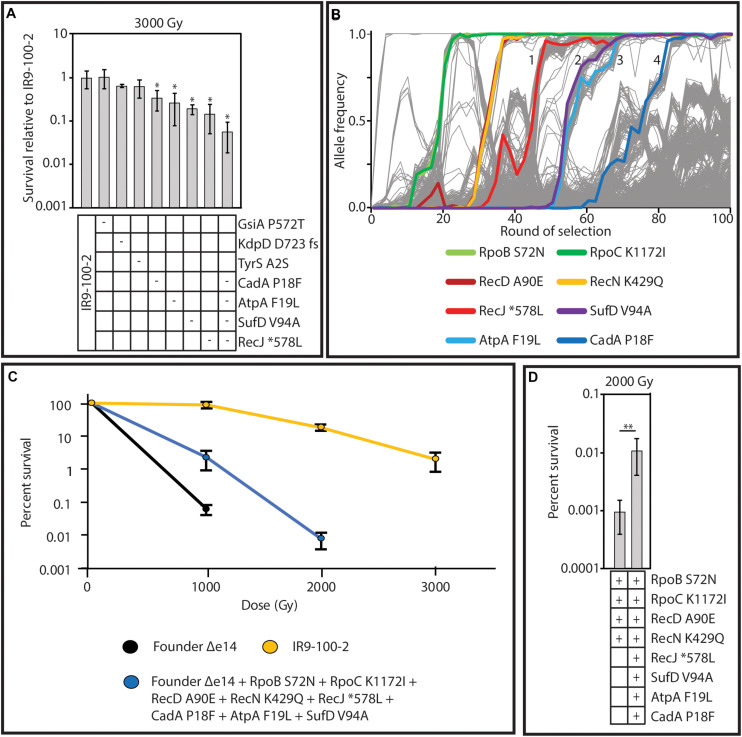
Mutations which contribute to IR resistance in lineage IR9. **(A)** Wild-type sequences of the gene encoding the indicated protein variants was moved from Founder Δe14 into IR9-100-2 as described in the section “Materials and Methods.” A ‘-’ symbol indicates that the variant was reverted to the wild-type allele in that strain. Each strain was assayed for IR resistance alongside biological triplicate of the parent strain (IR9-100-2) and the average percent survival of the experimental strains was compared to that of the parent strain. All strains were exposed to 3,000 Gy of electron beam IR and percent survival was determined via calculating CFU/mL before and after irradiation. Error bars represent the standard deviation of CFU/mL calculations of at least two independent experiments performed in biological triplicate. Statistical significance of percent survival relative to the parent strain was determined using a one-tailed Student’s *T* test. The ‘*’ symbol represents significant difference in survival compared to IR9-100-2 with a *p*-value < 0.01. **(B)** The frequency of the variants which enhance IR resistance in lineage IR9. The variants highlighted are those which were shown to be involved in IR resistance in panel A, or those that were previously discovered (Bruckbauer et al., 2019b). The selective sweeps after round 50 are indicated numerically. **(C)** The mutations which have been found to enhance IR resistance in lineage IR9 do not comprise the entirety of the IR-resistant phenotype of IR9-100-2. The indicated variants were placed in the Founder Δe14 genetic background as described in the section “Materials and Methods.” Founder Δe14 does not exhibit survival past 1,000 Gy, so no further doses were tested for this strain. No countable colonies were observed at a dose of 3,000 Gy for Founder Δe14 with the 8 indicated variants. **(D)** The indicated protein variants were moved from the appropriate evolved isolate to Founder Δe14 as described in the section “Materials and Methods.” A ‘ + ’ symbol indicates the presence of the variant. Each strain was assayed for survival at a dose of 2,000 Gy of electron beam IR. Percent survival was determined via calculating CFU/mL before and after irradiation. Error bars represent the standard deviation of CFU/mL calculations from a single experiment in biological triplicate. Statistical significance of percent survival between strains was determined using a one-tailed Student’s *T* test. The ‘**’ symbol represents significant difference in survival compared to IR9-100-2 with a *p*-value < 0.05.

Considering the mutational burden of IR9-100-2, with 806 mutations (excluding deletions and insertions; Supplementary Table S3), we hypothesized that placing single mutations in an otherwise wild-type genetic background (as done previously) may not sufficiently provide the genetic context necessary for mutations to measurably contribute to IR resistance. Therefore, to find mutations which enhance IR resistance, we converted mutant alleles to the wild-type base pair in the evolved isolate and assayed for a reduction in survival after irradiation. We focused on 5 genes with fixed alleles in at least two populations (*gsiA*, *kdpD*, *tyrS*, *atpA*, and *recJ)* and two genes with a fixed allele in IR9-100 with at least four prior mutations in the same gene (*sufD* and *cadA*). Of the seven candidates, four proved to have measurable effects on IR resistance. When converted to the wild type allele, variants of RecJ (^∗^578L; ‘^∗^’ indicating the stop codon of RecJ), AtpA (F19L), CadA (P18F), and SufD (V94A) each produced a significant reduction in IR resistance (Figure 11A). When all four of these variants were converted to wild-type sequences in combination, the reduction in IR resistance of this new version of IR9-100-2 was significantly greater than observed upon loss of any single mutant.

The variants of RecJ, SufD, AtpA and CadA help to define the four selective sweeps that have occurred in IR9 since round 50 of selection (Figure 11B). Therefore, we hypothesized that these four variants, combined with the variants identified that enhance IR resistance after 50 cycles of selection (RpoB S72N, RpoC K1172I, RecD A90E and RecN K429Q Bruckbauer et al., 2019b) contribute the majority of the evolved IR resistant phenotype of lineage IR9 at selection cycle 100. Combining these 8 variants in the otherwise wild-type strain Founder Δe14 significantly enhanced the IR resistance of *E. coli* but did not increase IR resistance to the level of IR9-100-2 (Figure 11C). However, a strain with the 8 identified variants was significantly more IR-resistant than Founder Δe14 containing only the four IR resistance-enhancing variants identified at round 50 of selection (Figure 11D). Therefore, while these 8 variants comprise a significant portion of the evolved IR-resistant phenotype of the IR9 lineage after 100 cycles of selection, the wild-type genetic background does not have the appropriate mutational context to produce the full IR-resistant phenotype of IR9-100-2. Additional and as yet unidentified contributions to the phenotype are present.

### Profile of Proteome Oxidation Suggests an IR-Resistance Mechanism Similar to *Deinoccocus radiodurans*

Many of the protein variants which enhance IR resistance that we have currently identified affect DNA repair enzymes (Harris et al., 2009; Byrne et al., 2014; Bruckbauer et al., 2019b; Figure 8). Such modifications are suggestive of the necessity to repair significant DNA damage after exposure to ionizing radiation. However, there is significant evidence that the highly IR-resistant bacterium *D. radiodurans* can survive extreme doses of IR in part due to an extraordinary ability to ameliorate intracellular ROS, which in turn protects essential cellular proteins from inactivating oxidation (Daly et al., 2004, 2010; Slade and Radman, 2011). In order to investigate this hypothesis further, we previously quantified the proteome oxidation induced by a lethal dose of IR (1,000 Gy) in *E. coli* utilizing mass spectrometry (Bruckbauer et al., 2020). Low levels (less than 2-fold increase) of IR-induced hydroxylation was observed across the *E. coli* proteome (158 total hydroxylated peptides), with the most hydroxylation clustering on a small number of proteins. In contrast to *E. coli*, the same dose of IR induced only a single hydroxylated peptide in *D. radiodurans*. This observation supports the previously published results of the innate ability of *D. radiodurans* to ameliorate ROS(Daly et al., 2004, 2010).

Lineage IR10 has evolved along a unique trajectory, where it is the only population to develop a fixed mutation in *recA* but not *recN* or *rpoB*, and it is the only lineage to not have lost the e14 prophage at early selection cycles (Bruckbauer et al., 2019b). Furthermore, population IR10-100 is the most IR resistant population after 100 cycles of selection (Figure 1). Therefore, we chose an isolate from this population (IR10-100-1) for analysis of total proteome oxidation after IR exposure utilizing the previously established mass spectrometry pipeline (Bruckbauer et al., 2020). The level of IR-induced hydroxylation was greatly reduced relative to wild type cells. It was also remarkably similar to that previously observed for *D. radiodurans*. Only one peptide exhibited significantly increased hydroxylation (Table 3). This peptide maps to the penicillin-binding protein activator LpoB, which was not a significant target of hydroxylation in the wild-type strain MG1655 (Bruckbauer et al., 2020). Notably, glyceraldehyde 3′-phosphate dehydrogenase (GAPDH), a major target of oxidation in wild type *E. coli* cells (Bruckbauer et al., 2020), was not noticeably oxidized in this evolved strain although the gene encoding GAPDH has not been altered. Given the sensitivity of the *E. coli* GAPDH to ROS generated by IR, this result strongly implies a greatly reduced level of ROS in the cytoplasm after irradiation, probably due to amelioration by cytoplasm components.

**TABLE 3:** IR-induced proteome oxidation in evolved isolate IR10-100-1.

**TMT-Mass spectrometry dataset**	**Total peptides quantified**	**No fold change (1000 Gy/0 Gy)**	**Fold change > 1 (1000 Gy/0 Gy)**	**Fold change > 2 (1000 Gy/0 Gy)**	**Fold change < 1 (1000 Gy/0 Gy)**	**Fold change < 0.5 (1000 Gy/0 Gy)**
MG1655* (1,938 proteins detected)						
Total peptides	13262	11703	764	48	795	2
Hydroxylated peptides (+ 16 Da)	1664	1437	175	19	52	0
D. radiodurans* (1,815 proteins detected)						
Total peptides	11526	11525	1	1	0	0
Hydroxylated peptides (+ 16 Da)	1779	1778	1	1	0	0
**IR10-100-1 (2,306 proteins detected)**						
Total peptides	23240	23239	1	1	0	0
Hydroxylated peptides (+ 16 Da)	3150	3149	1	1	0	0

*The MG1655 and *D. radiodurans* datasets were previously published (Bruckbauer et al., 2020). TMT-MS was performed and data was analyzed as described in the “MATERIALS AND METHODS” section.*

## Discussion

After 100 cycles of selection we have generated the most IR-resistant *Escherichia coli* populations to date. These populations now exhibit 100-fold higher survival at a dose of 3,000 Gy compared to the same lineages after 50 cycles of selection (Figure 1). Survival of wild type (founder) *E. coli* cells after 3,000 Gy is not readily measurable. Although fitness trade-offs have become apparent (Figures 2, 3), an upper limit to evolved IR resistance is not yet in sight. Building on DNA repair optimizations observed over the first 50 cycles of selection (Bruckbauer et al., 2019b), mutations driving evolution of IR resistance in lineage IR9 throughout the subsequent 50 selection cycles affect ATP synthesis (AtpA), Fe-S cluster protein repair (SufD), and cadaverine metabolism (CadA) in addition to further modification of DNA repair (RecJ). Substantial and as yet unidentified contributions to the phenotype are present in the genetic backgrounds present in the evolved populations.

Over an estimated 1,200 generations, the strong selection pressure used in this study has successfully generated extreme IR resistance in a naturally IR-sensitive organism. The Founder strain of *E. coli* used in this study does not survive IR doses exceeding 1,000 Gy (Figure 1B). Our experimentally evolved populations of *E. coli* now exhibit readily measurable survival at a dose as high as 4,000 Gy (Figure 1B). As cycles of selection have continued, the shoulder of IR resistance (where little to no death is observed) of these *E. coli* populations has begun to mirror that of the highly radioresistant bacterium *D. radiodurans*. *D. radiodurans* and two of the evolved populations exhibit little loss in viability at a dose of 2,000 Gy. Furthermore, at least one evolved isolate (IR10-100-1) is able to match *D. radiodurans*’ level of proteome protection from IR-induced protein hydroxylation (Table 3). The molecular basis of that proteome protection is under investigation.

Several observations demonstrate that our evolution protocol has produced IR-resistant specialists, rather than generalist bacteria capable of combating a broad spectrum of DNA-damaging agents or other challenges. First, isolates from each population at round 50 of selection (Bruckbauer et al., 2019b) and now round 100 of selection exhibit highly variable resistance to non-IR DNA damaging agents (Figure 3A). The IR-resistant phenotype does not automatically extend to any of these other agents. Second, isolates from round 100 of selection are not highly desiccation resistant (Figure 3B). Desiccation resistance is often correlated with extreme radiation resistance in nature (Rainey et al., 2005), as is likely the case with *D. radiodurans* (Tanaka et al., 2004; Fredrickson et al., 2008). Third, a high Mn to Fe ratio is another correlate of high IR resistance in natural bacterial isolates (i.e., increasing Mn:Fe ratio aligns with increasing IR resistance across species) (Daly et al., 2004, 2010; Sharma et al., 2017). However, we see no significant change in Mn or Fe levels in our evolved *E. coli* lineages (Supplementary Figure S1) even though we do document high levels of protection from protein oxidation. The results indicate the presence of high levels of ROS amelioration utilizing mechanisms that do not involve increases in the cellular level of Mn ions. All of these observations speak to the capacity of this directed evolution approach to elucidate mechanisms specific to IR resistance.

Our results suggest that while there is overlap between desiccation and IR resistance (Tanaka et al., 2004), our evolved isolates have developed mechanisms of IR resistance significantly different from those of naturally radioresistant organisms. The difference may be accounted for by the need of these *E. coli* populations to adapt to acute irradiation, rather than chronic IR exposure. A difference in the ability to survive acute versus chronic irradiation in natural and experimentally evolved IR-resistant isolates has been previously documented (Shuryak et al., 2019, 2017). As such, our evolved *E. coli* may not necessarily model how environmental IR resistance may develop, but rather how resistance to extreme doses of acute IR exposure can evolve, and to what extent. Alterations to the Mn/Fe ratio has been closely associated with IR resistance in nature (Daly, 2009; Daly et al., 2004; Sharma et al., 2017) but it is by no means the only mechanism that supports this extremophile phenotype (Cox and Battista, 2005). To date, no changes in the Mn/Fe ratio in our evolved strains has been detected, allowing us to elucidate additional mechanisms by which IR resistance can be achieved.

Adaptation to the niche of IR resistance has led to significant alteration of phenotypes and genotypes compared to the Founder strain of *E. coli*. We assayed two isolates from separate evolved populations (IR9-100 and IR10-100) for their response to DNA damage (the SOS response) and response to ROS stress (utilizing the promoter of the superoxide dismutase, *sodA*). Each exhibited altered SOS expression either in the absence of IR compared to the Founder (isolate IR9-100-2) or after irradiation with 1,000 Gy (isolate IR10-100-1) (Figure 4). Furthermore, these isolates exhibit a moderately modified response to ROS stress from IR compared to the Founder strain, but again each isolate is distinct. Constitutive activation of the *sodA* promoter is seen in IR9-100-2, and there is IR-insensitive *sodA* regulation in IR10-100-1 (Figure 5). Such results highlight that these populations have not only diverged significantly from wild-type *E. coli*, but each population has also diverged significantly from each other. As exhibited by their responses to ROS, IR9-100-2 may have adopted the strategy of a constitutive stress response to deal with the ROS generated by IR. In contrast, IR10-100-1 may be able to suppress ROS entirely, preventing activation of an ROS stress response. Such a hypothesis is supported by the level of proteome protection from IR-induced oxidation in IR10-100-1 (Table 3). These differences not only hint that there are multiple solutions to the problem of IR exposure, but that one solution may constrain or expand future evolvability of each population (i.e., ROS suppression precludes development of a constitutive ROS stress response phenotype).

The genotypes of each evolving population strongly support this divergent adaptation. Each evolved population differs in the numbers of polymorphisms (Table 1), the pattern of selective sweeps and clonal interference (Figures 6, 9), types of large genomic deletions and rearrangements detected (Figures 7, 8), and the candidate mutations driving IR resistance since round 50 of selection (Table 2).

Surprisingly, lineages IR9 and IR10 have each experienced a substantial deletion of over 100 kbp at nearly the same genetic locus (starting either after or before the insertion element within the *ydbA* gene, respectively). The deletions appear to be mediated by insertion elements. These are the only large deletions detected in any of the four populations, aside from loss of prophage (Figure 7). Approximately 100 predicted coding regions are lost in these deleted areas, although no deleted gene has a clear relation to IR resistance or sensitivity (Supplementary Table S2). It seems likely that such a significant deletion, ∼ 2% of the 4.6 Mbp *E. coli* genome, would affect genome replication or stability. However, defining such an effect is beyond the scope of this current work. Interestingly, insertion element-mediated deletions have been previously noted in this chromosomal region (Lee et al., 2016).

In addition to this deletion event, the downstream 65 kbp in IR9 is duplicated and inverted. This significant rearrangement occurred at the same time as the deletion in IR9 (round 28); there is no evidence that such a duplication also occurred in lineage IR10. Given the presence of an insertion element on the 5′ end of the duplicated region (present within the Qin prophage), which replaces the 3′ insertion element of the IR9 deletion, it is likely that the duplication is also insertion element mediated. Among the 79 duplicated coding regions are the Qin prophage, and the *terC* and *dif* genetic elements. Prophages, particularly Qin, can have significant effects on cell growth (Faubladier and Bouche, 1994), stress survival (Wang et al., 2010), and acquisition of novel gene functions (Ramisetty and Sudhakari, 2019). We have yet to characterize any effect from duplication of the Qin prophage in IR9. Given the significant effects of the action of these insertion elements on genome structure, further investigation on the effect of IR on insertion element (as well as prophage) expression and activity is warranted. In *D. radiodurans*, the insertion element ISDra2 appears to be activated by IR exposure due to increased ssDNA availability during DNA repair (Pasternak et al., 2010). However, this insertion element belongs to a different IS family than that which appears to be mediating the deletion and duplication events in our evolved populations.

The *terC* and *dif* genetic elements are important for termination of genome replication (Neylon et al., 2005; Carnoy and Roten, 2009), and the effect of duplication of these elements is unknown. Given the extreme fitness defects of a *dif* deletion and that the duplication is centered on *dif*, it is highly unlikely that the IR9 lineage could lose significant portions of the duplication through homologous recombination. Any such event would also delete both copies of *dif.* In the context of an evolution experiment, this permanent duplication event is particularly interesting. Gene duplication events often drive evolution of novel protein functions (Hughes, 1994; Blount et al., 2012). While there is no evidence for such an occurrence yet, selection cycles are ongoing and the duplication event in IR9 significantly increases the future evolvability of this lineage.

As our experimental evolution effort has continued, the complexity of each of these lineages has increased alongside IR resistance. Such diversity has not only led to each lineage diverging from one another but has also produced diversity within each lineage. Clonal interference has spawned long-term competing subpopulations in three of the four lineages (Figure 9). These subpopulations have coexisted since approximately round 60 of selection in IR9 and IR11, and since round 40 of selection in IR10. It is currently unclear if the relationship between each of these subpopulations is indeed competitive, or if stable coexistence is developing. However, it will be interesting to follow the trajectories of these populations and observe any link between the complexities of experimentally evolved populations exposed to IR and those in natural populations. Mixed populations of IR-resistant and sensitive microorganisms enhance IR resistance of the sensitive cells (Shuryak et al., 2017). A similar phenomenon may play a role in the evolved phenotypes of our populations where cells in the mixed population may be more resistant than a clonal isolate from that population.

In our early efforts to define the nature of these subpopulations, we have discovered that the yellow-brown colony phenotype of a subpopulation of lineage IR9 accumulates species of the heme precursor, porphyrin (Figure 10). On the surface, this porphyrin accumulating phenotype appears to be in direct opposition to extreme resistance to IR, as porphyrin accumulation from bacteria to humans is linked with visible light sensitivity (Miyamoto et al., 1991; Nishimura et al., 1995; Tatsumi and Wachi, 2008; Turlin et al., 2014). The genetic basis of this porphyrin accumulation is yet to be determined and the effect of this phenotype on IR resistance is not yet known.

**FIGURE 12:**
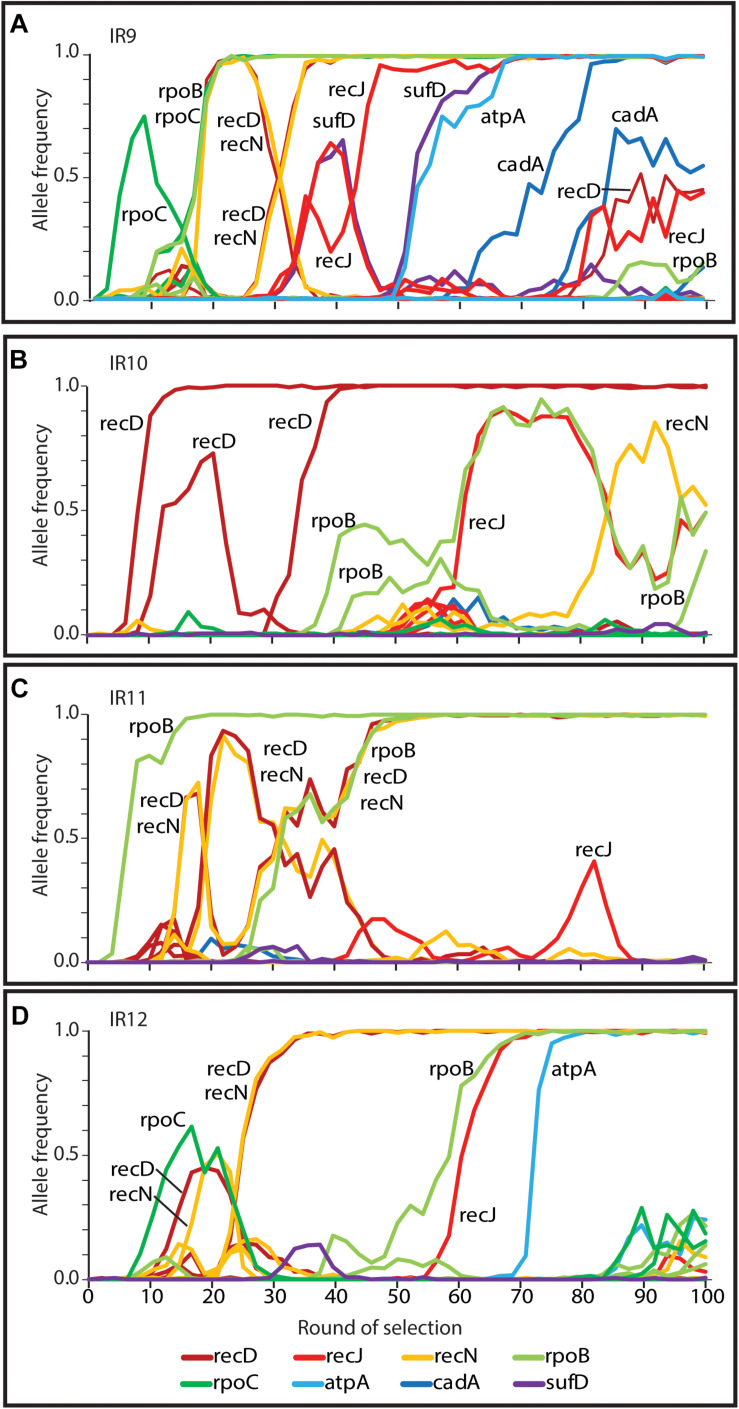
Mutations in genes linked to IR resistance in lineage **(A)** IR9 that are also present in **(B)** IR10, **(C)** IR11, and **(D)** IR12. Frequencies of mutations in genes implicated in evolved IR resistance (*rpoB*, *rpoC*, *recD*, *recN, recJ*, *sufD, atpA*, and *cadA*) are depicted. Both synonymous and non-synonymous mutations are included. These data indicate that genetic parallelism was highly prevalent in IR9, IR11, and IR12 prior to round 50 of selection. Over the next 50 cycles, parallel evolution was apparent in IR9 and IR12 (with high frequency variants of *recJ* and *atpA*). Only IR9 has a frequency allele of *cadA*, suggesting that the evolving populations are now diverging in mechanisms of IR resistance.

Due to extensive genetic parallelism prior to round 50 of selection, population IR9 provided a model to study the likely driving mutations of experimentally evolved IR resistance in the evolving lineages at round 50. Mutations identified which enhanced IR resistance in IR9-50 affected *recD*, *recN*, and *rpoB*/*C*; mutations in these genes were highly prevalent in three of the four populations. Since round 50 of selection, we have now identified alleles of *recJ*, *atpA*, *sufD*, and *cadA* which enhance IR resistance of population IR9-100 (Figure 11). While modification of the ssDNA exonuclease RecJ is suggestive of further optimization of DNA repair, alleles of *atpA*, *sufD*, and *cadA* represent novel drivers of IR resistance. Mutations in these genes are rare or non-existent in the remaining three populations. While alleles of these 8 genes (*rpoB, rpoC, recD, recN, recJ, atpA, sufD*, and *cadA*) appear often in IR9 and account for all 7 selective sweeps in this lineage, they account for only 2 of 6 sweeps in IR10 and IR11, and 4 of 5 sweeps in IR12 (Figure 12). This observation suggests that IR9 and IR12 may have traversed a similar fitness landscape to IR resistance, whereas the mutations which define IR resistance in IR9 do not explain the phenotypes of IR10 and IR11.

Only one of these newly identified variants, in RecJ, affects DNA repair machinery. RecJ is a 5′ - > 3′ exonuclease involved in homologous recombination (Lovett and Kolodner, 1989). The variant of RecJ (^∗^578L) in IR9-100 has the stop codon of the *recJ* gene mutated to a Leucine codon, adding 23 amino acids to the C terminus of the RecJ protein before the next stop codon. The effect that this mutation has on RecJ is unclear, although inactivation of RecJ cannot be ruled out. A previous transposon insertion library selected for survival after IR exposure did not identify RecJ as necessary for survival (Byrne et al., 2014). Interestingly, the RecJ protein of the highly radioresistant bacterium *D. radiodurans* contains a novel C terminal domain compared to that of *E. coli* (Cheng et al., 2015).

The remaining variants are the alpha subunit of the F_1_ ATP synthase subcomplex (AtpA), a lysine decarboxylase which promotes cadaverine biosynthesis (CadA), and iron-sulfur (Fe-S) cluster biogenesis (SufD). This is the first instance of modification to these pathways enhancing experimentally evolved IR resistance. The *E. coli* ATP synthase is comprised of the intermembrane proton pump F_0_ subcomplex, the catalytic ATP synthase F_1_ subcomplex, and the stator stalk which coordinates each sub complex (Weber, 2006). AtpA, the alpha subunit of the F1 subcomplex, forms a hexamer with three subunits of AtpB. The N-terminal 20 amino acids of AtpA has been shown to make important contacts with AtpH, the delta subunit of the stator stalk which is required for ATP synthase activity. The AtpA F19L isolated from lineage IR9 variant likely has a modified interaction with this stator stalk, as indicated on the crystal structure of ATP synthase (Supplementary Figure S4; **PDB: 5T4O** Sobti et al., 2016). The precise effect of altered stability of the ATP synthase complex on IR resistance is not known. A second fixed mutation in AtpA (G199D) is present in population IR12-100.

CadA is a pentamer of 5 dimer subunits and is involved in pH homeostasis, using protons to generate the polyamine species cadaverine through decarboxylation of free lysine residues (Kanjee et al., 2011). Polyamines have been previously implicated in IR resistance (Oh and Kim, 1998; Lee et al., 2019). It is unclear what effect the P18F mutation may have on CadA function. This mutation occurs at the interface between CadA subunits (Supplementary Figure S5; PDB: 3N75, Kanjee et al., 2011).

SufD is a component of the SufBC_2_D complex, which is responsible for *de novo* biogenesis of Fe-S clusters. Proteins containing an Fe-S cofactor are a clear target of ROS generated by IR (Imlay, 2006). Therefore modification of the machinery to generate Fe-S clusters may bolster cellular IR resistance. The precise role of SufD is unknown (Garcia et al., 2019), but this protein has been implicated in Fe acquisition for the SufBC_2_D complex (Saini et al., 2010). The V94A mutation is located in the N-terminal portion of SufD, an area with no known function or interactions (Hirabayashi et al., 2015). Mutations affecting the *sufD* or *cadA* genes are not apparent at high frequencies in any of the other three evolved populations, Multiple variants of SufD and CadA do appear at high frequencies in IR9 (Figure 12), and thus mutations affecting these proteins appear to be a prominent route toward fitness uniquely in IR9.

Our data thus-far suggest an early model of the trajectory of experimentally evolved IR resistance. After loss of the e14 prophage, which occurred in all four populations but only fixed in three, each population optimized DNA repair mechanisms (through mutations in *rpoB*/*C*, *recD* and *recN* in lineages IR9, IR11 and IR12; or mutations in *recA* and *recD* in IR10) (Bruckbauer et al., 2019b). After round 50 of selection, further modification to DNA repair through mutation of the ssDNA exonuclease RecJ was also apparent in three of the four populations. After this step, the four populations began to diverge significantly. Two populations, IR9 and IR12, exhibited a selective sweep involving a variant of AtpA. In IR9 a driving allele of *sufD* appeared concurrently with that in *atpA*. The final driving mutation identified in IR9-100 was a variant of CadA, which was unique to IR9. Given the number of selective sweeps in the remaining populations that are unaccounted for by the mutations listed above (Figure 12), and that we have not fully recapitulated the radioresistant phenotype of IR9-100-2 (Figure 11), there are many more mutations driving evolved IR resistance yet to be discovered. However, it is clear that after optimization of DNA repair, selection for IR resistance causes alterations to diverse aspects of cellular metabolism.

The original impetus to generate highly IR-resistant *E. coli* was to sequence these bacteria and determine what mutations underlie this phenotype (Harris et al., 2009; Byrne et al., 2014; Bruckbauer et al., 2019a). Now, we have developed an early roadmap for the evolution of IR-resistance far beyond what was previously generated (Bruckbauer et al., 2019b). What this roadmap suggests is that we may be able to identify mutations to enhance different aspects of IR resistance. For example, alterations in *recA* and *recJ* or complete inactivation of *recD* to enhance the cell’s capacity to deal specifically with IR-induced DNA strand breaks. While we do not yet understand the functional implications of variants of AtpA, CadA, and SufD on cell physiology and metabolism, it is unlikely these directly affect DNA repair. As we tease apart the functions of these mutations, we may be able to specifically enhance DNA repair capacity, or perhaps ROS amelioration and protein protection capacity as evidenced by the extreme protein protection of population IR10-100 (Table 3).

With this molecular toolbox of mutations, we may be able to engineer bacteria with mechanisms of IR resistance tailored to suit the task. Probiotics with enhanced DNA repair capacity may allow the gut or skin microbiome to withstand the damage caused by acute doses of IR delivered during cancer radiotherapy, whereas ROS ameliorating probiotics may be better suited to protect astronauts subjected to chronic IR stress during spaceflight. Such potential applications extend to cleanup of environmental radionuclide contamination and biotechnology. Furthermore, with a genetic basis for IR resistance, such a phenotype will he heritable and self-replicating; i.e., without the need of continuous application of an IR-mitigating agent. Given the presence of IR-resistance enhancing mutations in highly conserved genes such as *recA*, *rpoB*, and *atpA*, it may be possible to apply the lessons learned in *E. coli* to a wide range of bacteria. However, the complexity of the genotypes underlying this phenotype will make these goals challenging. Some of the phenotypic contributions we are beginning to see are unexpected and will require additional research to understand completely.

After 100 cycles of selection, these four *E. coli* lineages are approaching the extremophile IR resistant phenotype of *D. radiodurans*. Not only do our populations exhibit similar survival rates (Figure 1), but also enhanced protection of intracellular protein from IR-induced hydroxylation (Table 3). Although it has been previously shown that *E. coli* can achieve similar levels of resistance through the addition of ROS-ameliorating agents such as Mn (Daly et al., 2010), the resistance generated in this study is purely a cellular innovation which can, and likely will, be continuously optimized as experimental evolution continues. Cycles of selection are continuing, and there is not yet an indication that these cells have reached a plateau of IR resistance.

## Data Availability Statement

The original contributions presented in the study are included in the article/Supplementary Material, further inquiries can be directed to the corresponding author.

## Author Contributions

SB, BM, MV, IL, JL, JT, and EW designed and carried out experiments and analyzed data. SB, JM, BB, AL, and CP carried out DNA sequencing efforts and analyzed large DNA sequencing datasets. BM and MS carried out mass spectrometry experiments and analyzed large datasets. SB, MS, CP, and MC designed, directed, and oversaw project. SB and MC wrote the manuscript. All authors edited the manuscript.

## Conflict of Interest

The authors declare that the research was conducted in the absence of any commercial or financial relationships that could be construed as a potential conflict of interest.

## Footnotes

1 https://chorusproject.org/

2 https://www.ncbi.nlm.nih.gov/sra

3 http://broadinstitute.github.io/picard

4 sourceforge.net/projects/bbmap/

## Supplementary Material

FIGURE S1 Metals analysis of evolved *E. coli* isolates after 100 cycles of selection. Concentrations of trace metals were analyzed in early exponential phase cultures of the noted strains by the University of Wisconsin State Hygiene Laboratory of Hygiene Trace Element Research Laboratory. Raw concentrations of trace elements for each biological replicate for each strain was normalized to the CFU/mL of each culture. These data represent the results of biological triplicates for each strain listed.

FIGURE S2 The MMC-induced SOS response in evolved isolates. The growth curves and relative fluorescence units (raw fluorescence normalized to OD600) are depicted for **(A)** Founder Δe14, **(B)** IR9-100-2, and **(C)** IR10-100-1. The relative SOS response of each strain was assayed using the SOS-controlled promoter of the *recN* gene driving expression of GFP. Cultures of indicated strains were grown in LB medium overnight and then to early exponential phase as described in the section “Materials and Methods.” Cells from 1 mL of aliquot of each strain were pelleted, and resuspended in the indicated medium. One-hundred μL of each was then aliquoted into a 96-well plate and then incubated overnight in a Biotek Synergy H1 plate reader, with OD_600_ measurements and Ex:485nm/Em:513nm measurements taken automatically every 10 min as described in the section “Materials and Methods.” This experiment is representative of two independent experiments performed in biological triplicate; error bars represent the standard deviation of the biological triplicate.

FIGURE S3 Read length and location of Oxford Nanopore reads used to determine structure of 65 kb duplication in IR9-100. To confirm the presence of a duplication event in IR9 (as suggested by the buildup of Illumina sequencing reads shown in Figure 7A), we utilized Oxford Nanopore Sequencing. Genomic DNA was submitted to the University of Wisconsin – Madison Biotechnology Center for sequencing as described in the section “Materials and Methods.” Three contigs mapping to the duplicated region were sufficiently large enough to confirm the presence and structure of the duplication. The location of these reads is shown. The red regions shown in Contig B and Contig C do not align well to the indicated area, therefore the precise sequence of this region is not known. Start and end nucleotide positions listed reference the *E. coli* MG1655 genome (NCBI Genbank Accession number: U00096.3).

FIGURE S4 Location of AtpA F19L variant in *E. coli* ATP synthase. **(A)** ATP synthase of *E. coli*. The F_0_ subunit of ATP synthase is colored in green. The individual components of the F_1_ component are shown: γ subunit (AtpG), gold; ε subunit (AtpC), magenta; δ subunit (AtpH), teal; β subunit (AtpB), orange; α subunit (AtpA), slate blue. **(B)** AtpA F19L may have altered contacts with the δ subunit of the F_1_ component of ATP synthase. The amino acids highlighted in red are the F19 residue of AtpA mutated to a Leucine. The crystal structure of ATP synthase used was previously published (PDB: 5T4O) (Sobti et al., 2016).

FIGURE S5 Location of P18F mutation in *E. coli* CadA. **(A)** The lysine decarboxylase (CadA) of *E. coli*. CadA forms a pentamer; a single subunit is colored in slate blue. The white box highlights the location of the P18 residue, mutated to a Phenylalanine. **(B)** Magnified view of the P18 residue mutated to a phenylalanine. The disordered loop of CadA opposing the P18F residue is shown as a stick model to highlight a potential interaction. The crystal structure of CadA used was previously published (PDB: 3N75) (Kanjee et al., 2011).

TABLE S1 Strains used in this study. Citations: (Blattner et al., 1997; Harris et al., 2009).

TABLE S2 Duplicated and deleted genes and genetic elements in IR9-100 and IR10-100.

TABLE S3 Number of polymorphisms in sequenced isolates from each population at round 100. Isolates designated with a ‘-‘ symbol have not yet produced whole genome sequencing data.

## References

[B1] Almond P. R. Biggs P. J. Coursey B. M. Hanson W. F. Huq M. S. Nath R. (1999). AAPM’s TG-51 protocol for clinical reference dosimetry of high-energy photon and electron beams. *Med. Phys.* 26 1847–1870. 10.1118/1.598691 10505874

[B2] Blattner F. R. Plunkett G. R. Bloch C. A. Perna N. T. Burland V. Riley M. (1997). The complete genome sequence of *Escherichia coli* K-12. *Science* 277 1453–1474. 10.1126/science.277.5331.1453 9278503

[B3] Blount Z. D. Barrick J. E. Davidson C. J. Lenski R. E. (2012). Genomic analysis of a key innovation in an experimental *Escherichia coli* population. *Nature* 489 513–518. 10.1038/nature11514 22992527 PMC3461117

[B4] Boothby T. C. Tapia H. Brozena A. H. Piszkiewicz S. Smith A. E. Giovannini I. (2017). Tardigrades use intrinsically disordered proteins to survive desiccation. *Mol. Cell* 65 975.e5–984.e5. 10.1016/j.molcel.2017.02.018 28306513 PMC5987194

[B5] Bruckbauer S. T. Minkoff B. B. Yu D. Cryns V. L. Cox M. M. Sussman M. R. (2020). Ionizing radiation-induced proteomic oxidation in *Escherichia coli*. *Mol. Cell Proteom.* 19, 1375–1395. 10.1074/mcp.RA120.002092 32536603 PMC8015010

[B6] Bruckbauer S. T. Trimarco J. D. Henry C. Wood E. A. Battista J. R. Cox M. M. (2019a). A variant of the *Escherichia coli* anaerobic transcription factor FNR exhibiting diminished promoter activation function enhances ionizing radiation resistance. *PLoS One* 14:e0199482. 10.1371/journal.pone.0199482 30673695 PMC6343905

[B7] Bruckbauer S. T. Trimarco J. D. Martin J. Bushnell B. Senn K. A. Schackwitz W. (2019b). Experimental evolution of extreme resistance to ionizing radiation in *Escherichia coli* after 50 cycles of selection. *J. Bacteriol.* 201: e00784-18. 10.1128/JB.00784-18 PMC6436341 30692176

[B8] Byrne R. T. Klingele A. J. Cabot E. L. Schackwitz W. S. Martin J. A. Martin J. (2014). Evolution of extreme resistance to ionizing radiation via genetic adaptation of DNA Repair. *eLife* 3:e01322. 10.7554/eLife.01322 24596148 PMC3939492

[B9] Cao G. Zhang M. Miao J. Li W. Wang J. Lu D. (2015). Effects of X-ray and carbon ion beam irradiation on membrane permeability and integrity in Saccharomyces cerevisiae cells. *J. Radiat. Res.* 56 294–304. 10.1093/jrr/rru114 25599994 PMC4380059

[B10] Carnoy C. Roten C. A. (2009). The dif/Xer recombination systems in *proteobacteria*. *PLoS One* 4:e6531. 10.1371/journal.pone.0006531 19727445 PMC2731167

[B11] Cheng K. Zhao Y. Chen X. Li T. Wang L. Xu H. (2015). A Novel C-Terminal Domain of RecJ is Critical for Interaction with HerA in *Deinococcus radiodurans*. *Front. Microbiol.* 6:1302. 10.3389/fmicb.2015.01302 26648913 PMC4663267

[B12] Cox M. M. Battista J. R. (2005). *Deinococcus radiodurans* - The consummate survivor. *Nat. Rev. Microbiol.* 3 882–892. 10.1038/nrmicro1264 16261171

[B13] Cox R. Charles H. P. (1973). Porphyrin-accumulating mutants of *Escherichia coli*. *J. Bacteriol.* 113 122–132. 10.1128/jb.113.1.122-132.1973 4567136 PMC251610

[B14] Daly M. J. (2009). A new perspective on radiation resistance based on *Deinococcus radiodurans*. *Nat. Rev. Microbiol.* 7 237–245. 10.1038/nrmicro2073 19172147

[B15] Daly M. J. (2012). Death by protein damage in irradiated cells. *DNA Repair.* 11 12–21. 10.1016/j.dnarep.2011.10.024 22112864

[B16] Daly M. J. Gaidamakova E. K. Matrosova V. Y. Kiang J. G. Fukumoto R. Lee D. Y. (2010). Small-molecule antioxidant proteome-shields in *Deinococcus radiodurans*. *PLoS One* 5:e12570. 10.1371/journal.pone.0012570 20838443 PMC2933237

[B17] Daly M. J. Gaidamakova E. K. Matrosova V. Y. Vasilenko A. Zhai M. Venkateswaran A. (2004). Accumulation of Mn(II) in, *Deinococcus radiodurans* facilitates gamma-radiation resistance. *Science* 306 1025–1028. 10.1126/science.1103185 15459345

[B18] Datsenko K. A. Wanner B. L. (2000). One-step inactivation of chromosomal genes in *Escherichia coli* K-12 using PCR products. *Proc. Natl. Acad. Sci. U.S.A.* 97 6640–6645. 10.1073/pnas.120163297 10829079 PMC18686

[B19] Davies R. Sinskey A. J. (1973). Radiation-resistant mutants of *Salmonella* typhimurium LT2: development and characterization. *J. Bacteriol.* 113 133–144. 10.1128/jb.113.1.133-144.1973 4567137 PMC251611

[B20] Dietel W. Pottier R. Pfister W. Schleier P. Zinner K. (2007). 5-Aminolaevulinic acid (ALA) induced formation of different fluorescent porphyrins: a study of the biosynthesis of porphyrins by bacteria of the human digestive tract. *J. Photochem. Photobiol. B* 86 77–86. 10.1016/j.jphotobiol.2006.07.006 16973372

[B21] Ding H. Saer R. G. Beatty J. T. (2019). Porphyrin excretion resulting from mutation of a gene encoding a class I fructose 1,6-bisphosphate aldolase in Rhodobacter capsulatus. *Front. Microbiol.* 10:301. 10.3389/fmicb.2019.00301 30853951 PMC6395792

[B22] Dose K. Bieger-Dose A. Labusch M. Gill M. (1992). Survival in extreme dryness and DNA-single-strand breaks. *Adv. Space Res.* 12 221–229. 10.1016/0273-1177(92)90176-x 11538142

[B23] Earl A. M. Mohundro M. M. Mian I. S. Battista J. R. (2002). The IrrE protein of Deinococcus radiodurans R1 is a novel regulator of recA expression. *J. Bacteriol.* 184 6216–6224. 10.1128/jb.184.22.6216-6224.2002 12399492 PMC151961

[B24] Erdman I. Thatcher F. MacQueen K. (1961). Studies on the irradiation of microorganisms in relation to food preservation: II. *Irradiation resistant mutants*. *Can. J. Microbiol.* 7 207–215. 10.1139/m61-027 13697071

[B25] Faubladier M. Bouche J. P. (1994). Division inhibition gene dicF of *Escherichia coli* reveals a widespread group of prophage sequences in bacterial genomes. *J. Bacteriol.* 176 1150–1156. 10.1128/jb.176.4.1150-1156.1994 7508908 PMC205167

[B26] Fredrickson J. K. Li S. M. Gaidamakova E. K. Matrosova V. Y. Zhai M. Sulloway H. M. (2008). Protein oxidation: key to bacterial desiccation resistance? *ISME J* 2 393–403. 10.1038/ismej.2007.116 18273068

[B27] Garcia P. S. Gribaldo S. Py B. Barras F. (2019). The SUF system: an ABC ATPase-dependent protein complex with a role in Fe-S cluster biogenesis. *Res. Microbiol.* 170 426–434. 10.1016/j.resmic.2019.08.001 31419582

[B28] Giel J. L. Rodionov D. Liu M. Blattner F. R. Kiley P. J. (2006). IscR-dependent gene expression links iron-sulphur cluster assembly to the control of O2-regulated genes in *Escherichia coli*. *Mol. Microbiol.* 60 1058–1075. 10.1111/j.1365-2958.2006.05160.x 16677314

[B29] Harris D. R. Pollock S. V. Wood E. A. Goiffon R. J. Klingele A. J. Cabot E. L. (2009). Directed evolution of radiation resistance in *Escherichia coli*. *J. Bacteriol.* 191 5240–5252. 19502398 10.1128/JB.00502-09 PMC2725583

[B30] Harris D. R. Tanaka M. Saveliev S. V. Jolivet E. Earl A. M. Cox M. M. (2004). Preserving genome integrity: the DdrA protein of Deinococcus radiodurans R1. *PLoS Biol.* 2:e304. 10.1371/journal.pbio.0020304 15361932 PMC515370

[B31] Hirabayashi K. Yuda E. Tanaka N. Katayama S. Iwasaki K. Matsumoto T. (2015). Functional dynamics revealed by the structure of the SufBCD complex, a novel ATP-binding Cassette (ABC) protein that serves as a scaffold for iron-sulfur cluster biogenesis. *J. Biol. Chem.* 290 29717–29731. 10.1074/jbc.M115.680934 26472926 PMC4705970

[B32] Hughes A. L. (1994). The evolution of functionally novel proteins after gene duplication. *Proc. Biol. Sci.* 256 119–124. 10.1098/rspb.1994.0058 8029240

[B33] Imlay J. A. (2006). Iron-sulphur clusters and the problem with oxygen. *Mol. Microbiol.* 59 1073–1082. 10.1111/j.1365-2958.2006.05028.x 16430685

[B34] Kanjee U. Gutsche I. Alexopoulos E. Zhao B. El Bakkouri M. Thibault G. (2011). Linkage between the bacterial acid stress and stringent responses: the structure of the inducible lysine decarboxylase. *EMBO J.* 30 931–944. 10.1038/emboj.2011.5 21278708 PMC3049219

[B35] Kazmi S. A. Bukhari A. I. (1978). A mutant of *Escherichia coli* which accumulates large amounts of coproporphyrin. *Biochim. Biophys. Acta* 541 420–424. 10.1016/0304-4165(78)90201-5 352406

[B36] Kempner E. S. (2001). Effects of high-energy electrons and gamma rays directly on protein molecules. *J. Pharm. Sci.* 90 1637–1646. 10.1002/jps.1114 11745722

[B37] Kwon S. J. de Boer A. L. Petri R. Schmidt-Dannert C. (2003). High-level production of porphyrins in metabolically engineered *Escherichia coli*: systematic extension of a pathway assembled from overexpressed genes involved in heme biosynthesis. *Appl. Environ. Microbiol.* 69 4875–4883. 10.1128/aem.69.8.4875-4883.2003 12902282 PMC169110

[B38] Layer G. Verfurth K. Mahlitz E. Jahn D. (2002). Oxygen-independent coproporphyrinogen-III oxidase HemN from *Escherichia coli*. *J. Biol. Chem.* 277 34136–34142. 10.1074/jbc.M205247200 12114526

[B39] Lee C. Y. Su G. C. Huang W. Y. Ko M. Y. Yeh H. Y. Chang G. D. (2019). Promotion of homology-directed DNA repair by polyamines. *Nat. Commun.* 10:65. 10.1038/s41467-018-08011-1 PMC6325121 30622262

[B40] Lee H. Doak T. G. Popodi E. Foster P. L. Tang H. (2016). Insertion sequence-caused large-scale rearrangments in the genome of *Escherichia coli*. *Nucleic Acids Res.* 44 7109–7119. 10.1093/nar/gkw647 27431326 PMC5009759

[B41] Li H. Durbin R. (2009). Fast and accurate short read alignment with Burrows-Wheeler transform. *Bioinformatics* 25 1754–1760. 10.1093/bioinformatics/btp324 19451168 PMC2705234

[B42] Lovett S. T. Kolodner R. D. (1989). Identification and purification of a single-stranded-DNA-specific exonuclease encoded by the recJ gene of *Escherichia coli*. *Proc. Natl. Acad. Sci. U.S.A.* 86 2627–2631. 10.1073/pnas.86.8.2627 2649886 PMC286970

[B43] Miller J. H. (1992). *A Short Course in Bacterial Genetics: A Laboratory Manual and Handbook for Escherichia coli* and Related Bacteria. New York, NY: Cold Spring Harbor Laboratory.

[B44] Miyamoto K. Nakahigashi K. Nishimura K. Inokuchi H. (1991). Isolation and characterization of visible light-sensitive mutants of *Escherichia coli* K12. *J. Mol. Biol.* 219 393–398. 10.1016/0022-2836(91)90180-e 2051480

[B45] Neylon C. Kralicek A. V. Hill T. M. Dixon N. E. (2005). Replication termination in *Escherichia coli*: structure and antihelicase activity of the Tus-Ter complex. *Microbiol. Mol. Biol. Rev.* 69 501–526. 10.1128/MMBR.69.3.501-526.2005 16148308 PMC1197808

[B46] Nishimura K. Nakayashiki T. Inokuchi H. (1995). Cloning and identification of the hemG gene encoding protoporphyrinogen oxidase (PPO) of *Escherichia coli* K-12. *DNA Res.* 2 1–8. 10.1093/dnares/2.1.1 7788523

[B47] Oh T. J. Kim I. G. (1998). Polyamines protect against DNA strand breaks and aid cell survival against irradiation in *Escherichia coli*. *Biotechnol. Tech.* 12 755–758. 10.1023/A:1008864618091

[B48] Parisi A. Antoine A. (1974). Increased radiation resistance of vegetative Bacillus pumilus. *Appl. Microbiol.* 28 41–46. 10.1128/aem.28.1.41-46.1974 4844266 PMC186583

[B49] Pasternak C. Ton-Hoang B. Bailone A. Chandler M. Sommer S. (2010). Irradiation-induced Deinococcus radiodurans genome fragmentation triggers transposition of a single resident insertion sequence. *PLoS Genet.* 6:e1000799. 10.1371/journal.pgen.1000799 20090938 PMC2806898

[B50] Rainey F. A. Ray K. Ferreira M. Gatz B. Z. Nobre M. F. Bagaley D. (2005). Extensive diversity of ionizing-radiation-resistant bacteria recovered from Sonoran Desert soil and description of nine new species of the genus Deinococcus obtained from a single soil sample. *Appl. Environ. Microbiol.* 71 5225–5235. 10.1128/AEM.71.9.5225-5235.2005 16151108 PMC1214641

[B51] Ramisetty B. C. M. Sudhakari P. A. (2019). Bacterial ‘grounded’ prophages: hotspots for genetic renovation and innovation. *Front. Genet.* 10:65. 10.3389/fgene.2019.00065 30809245 PMC6379469

[B52] Reisz J. A. Bansal N. Qian J. Zhao W. Furdui C. M. (2014). Effects of ionizing radiation on biological molecules–mechanisms of damage and emerging methods of detection. *Antioxid. Redox. Signal.* 21 260–292. 10.1089/ars.2013.5489 24382094 PMC4060780

[B53] Ruiz de Almodovar J. M. Bush C. Peacock J. H. Steel G. G. Whitaker S. J. McMillan T. J. (1994). Dose-rate effect for DNA damage induced by ionizing radiation in human tumor cells. *Radiat. Res.* 138 (1 Suppl.), S93–S96. 10.2307/3578771 8146338

[B54] Saini A. Mapolelo D. T. Chahal H. K. Johnson M. K. Outten F. W. (2010). SufD and SufC ATPase activity are required for iron acquisition during in vivo Fe-S cluster formation on SufB. *Biochemistry* 49 9402–9412. 10.1021/bi1011546 20857974 PMC3004146

[B55] Sakano K. Oikawa S. Hasegawa K. Kawanishi S. (2001). Hydroxyurea induces site-specific DNA damage via formation of hydrogen peroxide and nitric oxide. *JPN J. Cancer Res.* 92 1166–1174. 10.1111/j.1349-7006.2001.tb02136.x 11714440 PMC5926660

[B56] Santos A. L. Oliveira V. Baptista I. Henriques I. Gomes N. C. Almeida A. (2013). Wavelength dependence of biological damage induced by UV radiation on bacteria. *Arch. Microbiol.* 195 63–74. 10.1007/s00203-012-0847-5 23090570

[B57] Selvam K. Duncan J. R. Tanaka M. Battista J. R. (2013). DdrA, DdrD, and PprA: components of UV and mitomycin C resistance in Deinococcus radiodurans R1. *PLoS One* 8:e69007. 10.1371/journal.pone.0069007 23840905 PMC3698191

[B58] Sezonov G. Joseleau-Petit D. D’Ari R. (2007). *Escherichia coli* physiology in Luria-Bertani broth. *J. Bacteriol.* 189 8746–8749. 10.1128/JB.01368-07 17905994 PMC2168924

[B59] Sharma A. Gaidamakova E. K. Grichenko O. Matrosova V. Y. Hoeke V. Klimenkova P. (2017). Across the tree of life, radiation resistance is governed by antioxidant Mn(2+), gauged by paramagnetic resonance. *Proc. Natl. Acad. Sci. U.S.A.* 114 E9253–E9260. 10.1073/pnas.1713608114 29042516 PMC5676931

[B60] Shuryak I. Matrosova V. Y. Gaidamakova E. K. Tkvac R. Grichenko O. Klimenkova P. (2017). Microbial cells can cooperate to resist high-level chronic ionizing radiation. *PLoS One* 12:e0189261. 10.1371/journal.pone.0189261 29261697 PMC5738026

[B61] Shuryak I. Tkavc R. Matrosova V. Y. Volpe R. P. Grichenko O. Klimenkova P. (2019). Chronic gamma radiation resistance in fungi correlates with resistance to chromium and elevated temperatures, but not with resistance to acute irradiation. *Sci. Rep.* 9:11361. 10.1038/s41598-019-47007-9 31388021 PMC6684587

[B62] Slade D. Radman M. (2011). Oxidative Stress Resistance in *Deinococcus radiodurans*. *Microbiol. Mol. Biol. R.* 75 133–191. 10.1128/mmbr.00015-10 21372322 PMC3063356

[B63] Sobti M. Smits C. Wong A. S. Ishmukhametov R. Stock D. Sandin S. (2016). Cryo-EM structures of the autoinhibited E. *coli ATP synthase in three rotational states*. *eLife* 5:e21598. 10.7554/eLife.21598 28001127 PMC5214741

[B64] Tanaka M. Earl A. M. Howell H. A. Park M. J. Eisen J. A. Peterson S. N. (2004). Analysis of Deinococcus radiodurans’s transcriptional response to ionizing radiation and desiccation reveals novel proteins that contribute to extreme radioresistance. *Genetics* 168 21–33. 10.1534/genetics.104.029249 15454524 PMC1448114

[B65] Tatsumi R. Wachi M. (2008). TolC-dependent exclusion of porphyrins in *Escherichia coli*. *J. Bacteriol.* 190 6228–6233. 10.1128/JB.00595-08 18641137 PMC2546788

[B66] Turlin E. Heuck G. Simoes Brandao M. I. Szili N. Mellin J. R. Lange N. (2014). Protoporphyrin (PPIX) efflux by the MacAB-TolC pump in *Escherichia coli*. *Microbiologyopen* 3 849–859. 10.1002/mbo3.203 25257218 PMC4263509

[B67] Wang X. Kim Y. Ma Q. Hong S. H. Pokusaeva K. Sturino J. M. (2010). Cryptic prophages help bacteria cope with adverse environments. *Nat. Commun.* 1:147. 10.1038/ncomms1146 21266997 PMC3105296

[B68] Warming S. Costantino N. Court D. L. Jenkins N. A. Copeland N. G. (2005). Simple and highly efficient BAC recombineering using gaIK selection. *Nucleic Acids Res.* 33:e36. 10.1093/nar/gni035 15731329 PMC549575

[B69] Weber J. (2006). ATP synthase: subunit-subunit interactions in the stator stalk. *Biochim. Biophys. Acta* 1757 1162–1170. 10.1016/j.bbabio.2006.04.007 16730323 PMC1785291

[B70] Witkin E. M. (1946). Inherited differences in sensitivity to radiation in *Escherichia coli*. *Proc. Natl. Acad. Sci. U.S.A.* 32 59–68. 10.1073/pnas.32.3.59 16578194 PMC1078880

