# Corrigendum: Physiology of Highly Radioresistant *Escherichia coli* After Experimental Evolution for 100 Cycles of Selection

**DOI:** 10.3389/fmicb.2020.617806

**Published:** 2020-12-11

**Authors:** Steven T. Bruckbauer, Joel Martin, Benjamin B. Minkoff, Mike T. Veling, Illissa Lancaster, Jessica Liu, Joseph D. Trimarco, Brian Bushnell, Anna Lipzen, Elizabeth A. Wood, Michael R. Sussman, Christa Pennacchio, Michael M. Cox

**Affiliations:** ^1^Department of Biochemistry, University of Wisconsin-Madison, Madison, WI, United States; ^2^DOE Joint Genome Institute, Berkeley, CA, United States; ^3^Center for Genomic Science Innovation, University of Wisconsin School of Medicine and Public Health, Madison, WI, United States; ^4^Department of Systems Biology, Harvard Medical School, Boston, MA, United States

**Keywords:** ionizing radiation, experimental evolution, reactive oxygen species, double-strand breaks, DNA Repair, *Escherichia coli*, *Deinococcus radiodurans*

In the original article, there was a mistake in Table 1 as published. Three rows in Table 1 were inadvertently labeled incorrectly. The rows are in the “Transversions” category as follows: GC to TA, AT to TA, and GC to CG. We have re-labeled these rows so that these data under each population heading now reflects the correct number of each mutation type (in order, these rows are now AT to TA, GC to CG, and GC to TA). The corrected Table 1 appears below.

**Table 1:** Number and types of mutations in evolved populations after 100 cycles of selection.

	**Parent bp**	**Mutant bp**	**IR9-100**	**IR10-100**	**IR11-100**	**IR12-100**
Transitions	AT	GC	275	257	109	170
	GC	AT	445	376	244	484
Transversions	AT	CG	63	48	26	55
	AT	TA	196	139	79	186
	GC	CG	75	78	42	91
	GC	TA	350	267	173	348
Totals	Total		1404	1165	673	1334
	Transitions		720	633	353	654
	Transversions		684	532	320	680
Coding	Ts:Tv		1.05	1.19	1.10	0.96
	Synonymous		356	311	171	317
	Non-synonymous		822	669	383	813
	Stop gained		52	36	24	46
	Stop lost		3	2	1	1
	Start lost		3	2	0	2
	Insertions	+1	3	5	3	3
		+2	1	0	0	0
		+6	0	0	1	0
		+12	0	0	1	0
	Deletions	−1	137	79	32	65
		−2	4	1	2	3
		−3	1	0	0	2
		−11	0	1	0	0
		−29	1	0	0	0
		−39	0	0	1	0
	Total		1383	1106	619	1252
Non-coding	SNPs		189	158	100	169
	Insertions	+1	0	0	0	1
	Deletions	−1	15	11	5	12
		−2	0	0	0	1
Allele frequencies	Total		204	169	105	183
	Fixed (>99%)		332	95	164	306
	>50%		516	279	336	336
	>10%		1087	850	543	933
	>2%		1668	1260	732	1469

The authors apologize for this error and state that this does not change the scientific conclusions of the article in any way. The original article has been updated.

