# Supplementary figures and images for: Physiology of Highly Radioresistant Escherichia coli After Experimental Evolution for 100 Cycles of Selection
Source: Front Microbiol. 2020 Sep 22;11:582590. doi: 10.3389/fmicb.2020.582590 (PMC7536353; doi:10.3389/fmicb.2020.582590)

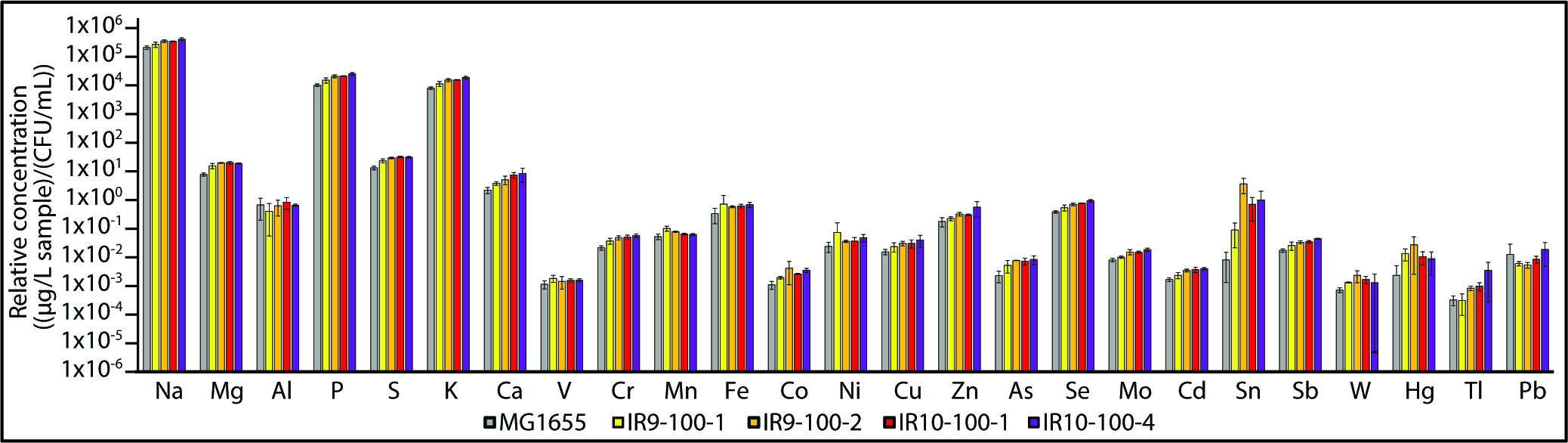

Supplement: FIGURE S1 — Metals analysis of evolved E. coli isolates after 100 cycles of selection. Concentrations of trace metals were analyzed in early exponential phase cultures of the noted strains by the University of Wisconsin State Hygiene Laboratory of Hygiene Trace Element Research Laboratory. Raw concentrations of trace elements for each biological replicate for each strain was normalized to the CFU/mL of each culture. These data represent the results of biological triplicates for each strain listed. [file Image_1.TIF]

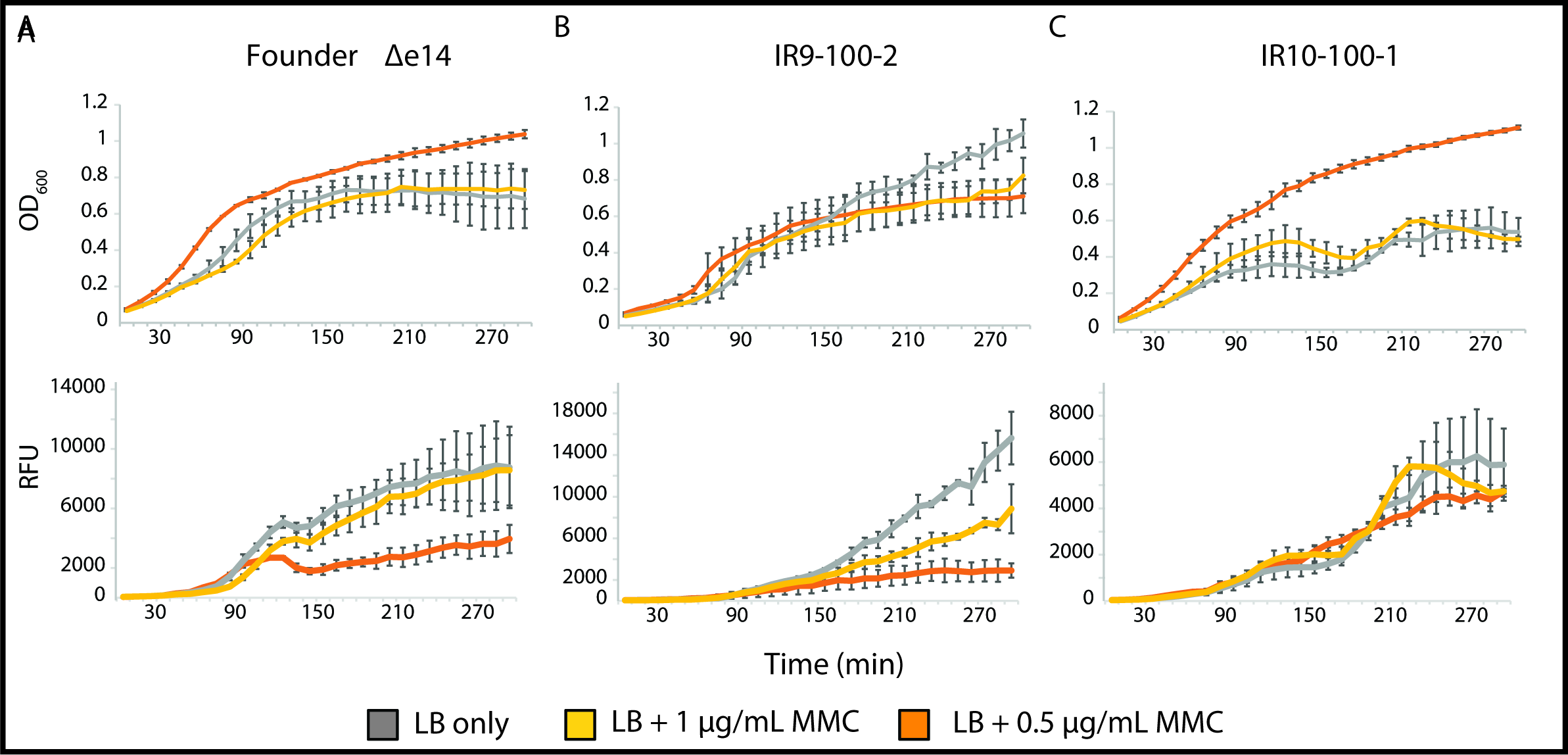

Supplement: FIGURE S2 — The MMC-induced SOS response in evolved isolates. The growth curves and relative fluorescence units (raw fluorescence normalized to OD600) are depicted for (A) Founder Δe14, (B) IR9-100-2, and (C) IR10-100-1. The relative SOS response of each strain was assayed using the SOS-controlled promoter of the recN gene driving expression of GFP. Cultures of indicated strains were grown in LB medium overnight and then to early exponential phase as described in the section “Materials and Methods.” Cells from 1 mL of aliquot of each strain were pelleted, and resuspended in the indicated medium. One-hundred μL of each was then aliquoted into a 96-well plate and then incubated overnight in a Biotek Synergy H1 plate reader, with OD600 measurements and Ex:485nm/Em:513nm measurements taken automatically every 10 min as described in the section “Materials and Methods.” This experiment is representative of two independent experiments performed in biological triplicate; error bars represent the standard deviation of the biological triplicate. [file Image_2.TIF]

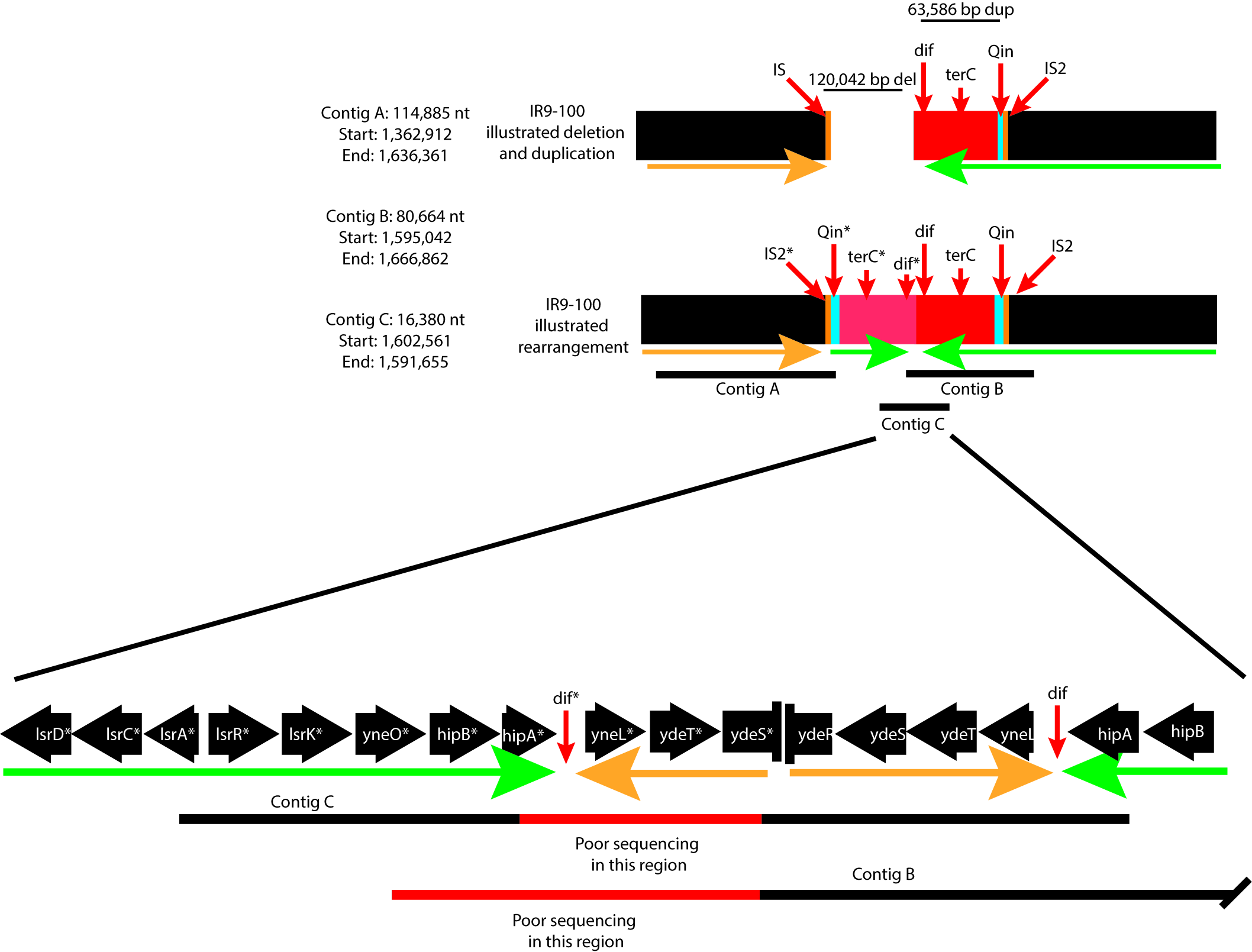

Supplement: FIGURE S3 — Read length and location of Oxford Nanopore reads used to determine structure of 65 kb duplication in IR9-100. To confirm the presence of a duplication event in IR9 (as suggested by the buildup of Illumina sequencing reads shown in Figure 7A), we utilized Oxford Nanopore Sequencing. Genomic DNA was submitted to the University of Wisconsin – Madison Biotechnology Center for sequencing as described in the section “Materials and Methods.” Three contigs mapping to the duplicated region were sufficiently large enough to confirm the presence and structure of the duplication. The location of these reads is shown. The red regions shown in Contig B and Contig C do not align well to the indicated area, therefore the precise sequence of this region is not known. Start and end nucleotide positions listed reference the E. coli MG1655 genome (NCBI Genbank Accession number: U00096.3). [file Image_3.TIF]

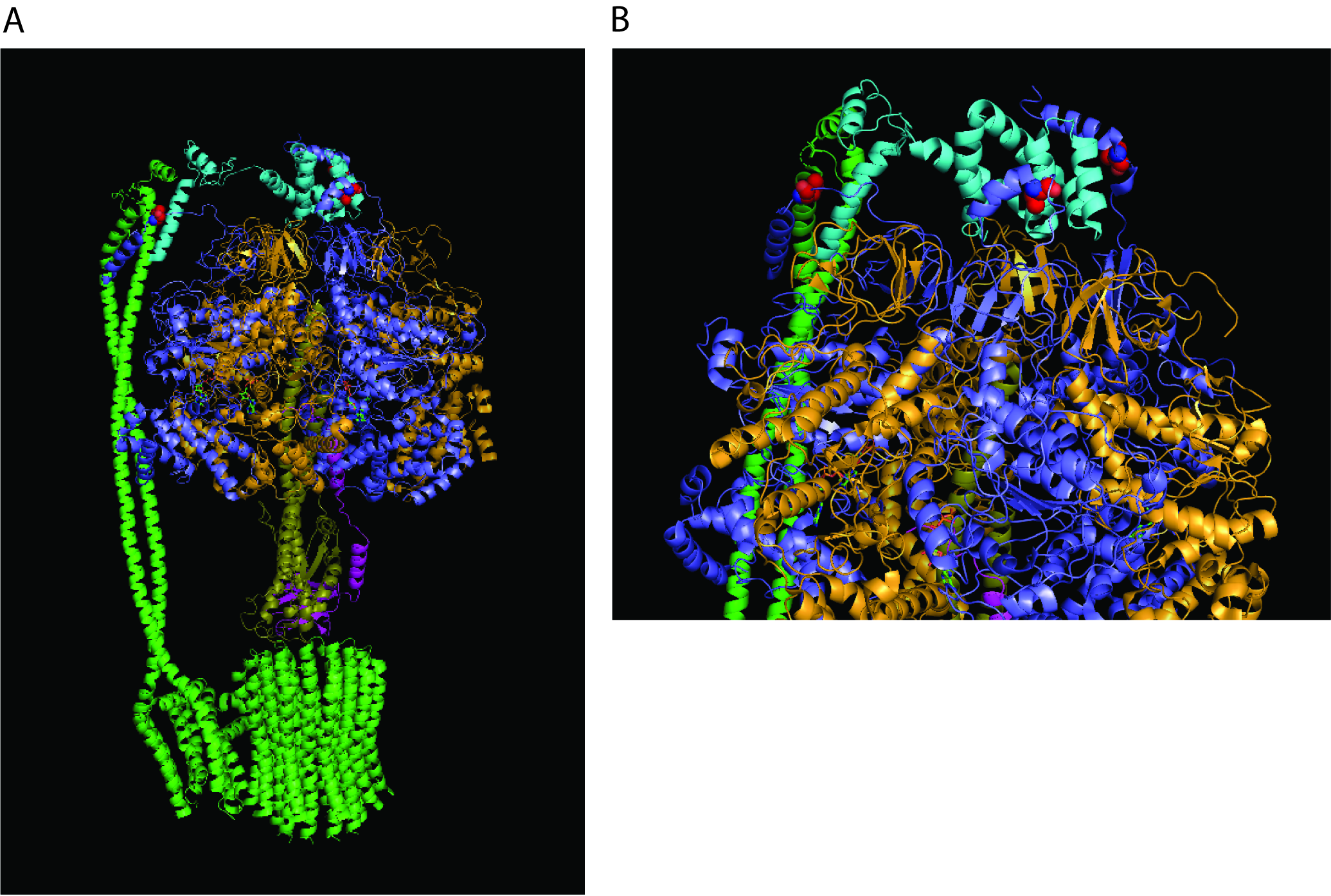

Supplement: FIGURE S4 — Location of AtpA F19L variant in E. coli ATP synthase. (A) ATP synthase of E. coli. The F0 subunit of ATP synthase is colored in green. The individual components of the F1 component are shown: γ subunit (AtpG), gold; ε subunit (AtpC), magenta; δ subunit (AtpH), teal; β subunit (AtpB), orange; α subunit (AtpA), slate blue. (B) AtpA F19L may have altered contacts with the δ subunit of the F1 component of ATP synthase. The amino acids highlighted in red are the F19 residue of AtpA mutated to a Leucine. The crystal structure of ATP synthase used was previously published (PDB: 5T4O) (Sobti et al., 2016). [file Image_4.TIF]

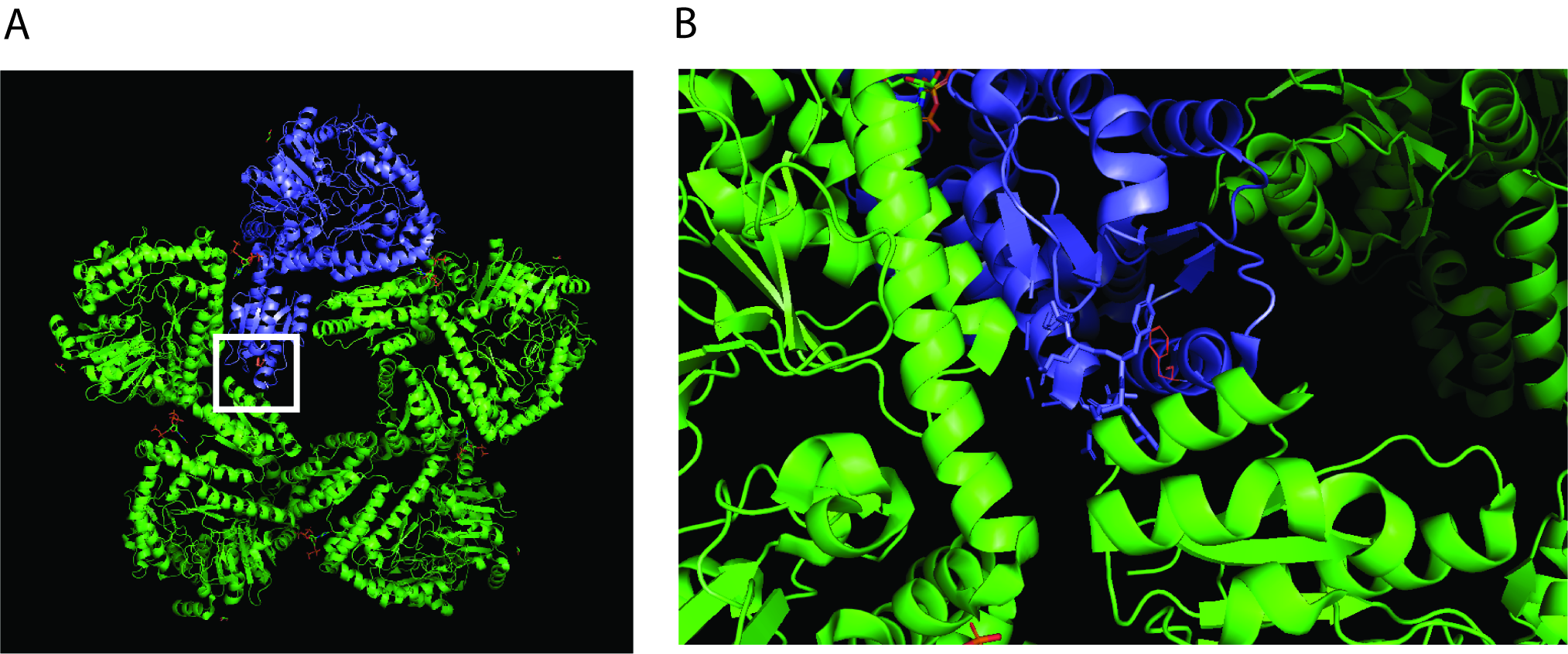

Supplement: FIGURE S5 — Location of P18F mutation in E. coli CadA. (A) The lysine decarboxylase (CadA) of E. coli. CadA forms a pentamer; a single subunit is colored in slate blue. The white box highlights the location of the P18 residue, mutated to a Phenylalanine. (B) Magnified view of the P18 residue mutated to a phenylalanine. The disordered loop of CadA opposing the P18F residue is shown as a stick model to highlight a potential interaction. The crystal structure of CadA used was previously published (PDB: 3N75) (Kanjee et al., 2011). [file Image_5.TIF]
